# Supplementary material for: A mathematical framework for understanding the spontaneous emergence of complexity applicable to growing multicellular systems
Source: PLoS Comput Biol. 2024 Jun 5;20(6):e1011882. doi: 10.1371/journal.pcbi.1011882 (PMC11182560; doi:10.1371/journal.pcbi.1011882)
Supplement: S1 Appendix — (DOCX) [file pcbi.1011882.s001.docx]

**APPENDIX S1.** **Detailed description of the division-decision model and the experimental data set**

Contents

[Detailed description of the division-decision model 2](#_Toc165993511)

[Other forms of dynamical equations under different regulatory logics 2](#_Toc165993512)

[The continuous model of the division-decision system 2](#_Toc165993513)

[Bi-stability of gene A in continuous model 3](#_Toc165993514)

[Ordinary differential equations that characterize the genetic network T1 in the continuous model 5](#_Toc165993515)

[Stochastic differential equations 7](#_Toc165993516)

[The derivation from the genetic network T1 to the genetic network T6 7](#_Toc165993517)

[Parameter values in the continuous model 9](#_Toc165993518)

[The booleanization of gene expression profiles and the identification of the cell state in *C.elegans* early development 13](#_Toc165993519)

# Detailed description of the division-decision model

## Other forms of dynamical equations under different regulatory logics

In addition to Equation 5, we also simulated the system using other gene regulatory logics and updating functions [42, 43], such as the traditional AND-gate, OR-gate, etc. In the single-gene system, we discovered another genetic network, O2, by providing each gene a constitutive expression additional to regulation (Equation *S*1 Figure S3). The network O2 allows the division-decision system to attain 1.0 bit of positional information at stable states (Figure S3b and S3c), with just one regulatory edge as the intercellular lateral inhibition. Similar to the O1-regulated system, the contacting state (*CS*) at step 7 provides the system with 1.0 bit of potential information, which is then transformed to 1.0 bit of positional information at step 8 by the rules prescribed by the genetic network O2 (Figure S3e).

In the two-gene system, using Equation *S*1 (Figure S3a), we discovered three genetic networks (T2, T3, and T4) that enabled controlled systems to attain multicellular stable states with 2.0 bit positional information (Figure S5). In T2, positional information increases in a way similar to that of T1 (Figure S5a). The T3-controlled system first attained unstable cell states with 2.0 bit of positional information, then transitioned from unstable to stable states with a reduction in positional information, and eventually reached multicellular stable states with 2.0 bit positional information (Figure S5b). In the T4-controlled system, positional information expanded to 2.0 bit through two steps: the positional information increased from 1.0 bit to 1.5 bit by updating the states of two cells, and then from 1.5 bit to 2.0 bit by changing two extra cells’ states (Figure S5c). Overall, they attained additional positional information by extracting potential information in the concatenated input matrix ([*S*, *CS*]).

## The continuous model of the division-decision system

We investigated the continuous version of the division-decision system. The network O1 contains two kinds of interactions: intracellular activation and intercellular lateral inhibition. We used the Hill function to describe these interactions and integrated them in two different ways: multiplication (AND-gate, Equation *S*2, Figure 4a) and addition (OR-gate, Equation *S*3, Figure 4b).

$$\frac{dA}{dt}=K_{selfA\_ac}\times\frac{A^{n}}{A^{n}+k_{selfA\_ac}^{n}}\times K_{AexA\_in}\times\frac{k_{AexA\_in}^{n}}{{Aex}^{n}+k_{AexA\_in}^{n}}-d_{A}\times A (Equation S2)$$

$$\frac{dA}{dt}=K_{selfA\_ac}\times\frac{A^{n}}{A^{n}+k_{selfA\_ac}^{n}}+K_{AexA\_in}\times\frac{k_{AexA\_in}^{n}}{{Aex}^{n}+k_{AexA\_in}^{n}}-d_{A}\times A (Equation S3)$$

Also, we tested transforming gene A's intracellular self-activation to constitutive expression, and the first term of the equation became a control parameter, K_constitutive_ (Figure 4c):

$$\frac{dA}{dt}=K_{constitutive}+K_{AexA\_in}\times\frac{k_{AexA\_in}^{n}}{{Aex}^{n}+k_{AexA\_in}^{n}}-d_{A}\times A (Equation S4)$$

## Bi-stability of gene A in continuous model

Using a Boolean model, we screened the genetic networks O1 (Figure 1d) and T1 (Figure 2c). Equation *S*2 had been used to regulate the expression of genes in the genetic networks, and the form of Equation *S*2 determined the bistable expression of gene A in both the genetic networks O1 and T1.

In the single-gene system, the expression of gene A in the genetic networks O1 (Figure 1d) and O2 (Figure S3b) could be described by Equations *S*2-*S*4, when transforming discrete gene expression to continuous gene expression. In one-dimensional division-decision systems, the simulation of the continuous model leads to increased positional information (Figure 4a, 4b and 4c). However, we found that only gene A described in Equation *S*2 was bistable. Under Equation *S*2, gene A exhibited bi-stability within a specific range of inhibition strength (Figure S9b), while Equation *S*3 had only one stable state of gene A, with the expression level determined by the intensity of extracellular inhibition (Figure S9d). When the regulatory logic for gene A is AND-gate, and the system has reached the 8-cell stage (generation 4), the 4 cells on each side of the border are stable in a high-expression state for gene A, but the 4 cells in the center of the system are stable in a low-expression state for gene A. When the regulatory logic of gene A is OR-gate, the system has reached to the 8-cell stage (generation 4), and the expression of gene A is alternately arranged between high and low states beginning from the cells at both ends of the border. The bi-stability of gene A affects the two distinct expression patterns. Through parameter scanning (1000 sets of parameters), we found that in systems where gene A's regulatory logic is the AND-gate, the gene expression pattern shown in Figure 4a will appear in the 8-cell stage if the parameter's value leads to gene A having bi-stability (77 sets of parameters); on the other hand, the pattern shown in Figure 4b will appear if the parameter's value leads to gene A does not have bi-stability (36 sets of parameters). Because gene A is not bistable in systems where its regulatory logic is the OR-gate, only the gene expression pattern seen in Figure 4b appears in the 8-cell stage (233 sets of parameters).

When the change rate of gene A over time was equal to zero, Equation *S*2 transformed into Equation *S*5:

$$K_{selfA\_ac}\times\frac{A^{n}}{A^{n}+k_{selfA\_ac}^{n}}\times K_{AexA\_in}\times\frac{k_{AexA\_in}^{n}}{{Aex}^{n}+k_{AexA\_in}^{n}}-d_{A}\times A=0 (Equation S5)$$

The intersection of curves corresponding to Equations *S*6 and *S*7 was the solution of variable A in Equation *S*5:

$$f\left( A \right)=d_{A}\times A (Equation S6)$$

$$g\left( A \right)=K_{selfA\_ac}\times\frac{A^{n}}{A^{n}+k_{selfA\_ac}^{n}}\times K_{AexA\_in}\times\frac{k_{AexA\_in}^{n}}{{Aex}^{n}+k_{AexA\_in}^{n}} (Equation S7)$$

In the phase portrait, Equation *S*6 corresponded to black line, while Equation *S*7 with different parameters (Aex) corresponded to dotted curves in various colors. Fixed points were found at the intersections of curves and the straight line: stable fixed points were found on the upper and lower sides of curves; unstable fixed points were found in the center of curves (Figure S9a). The results revealed that the gene A described in Equation *S*2 was bistable.

For the Equation *S*3, when the change rate of gene A over time was equal to zero, Equation *S*3 transformed into Equation *S*8:

$$K_{selfA\_ac}\times\frac{A^{n}}{A^{n}+k_{selfA\_ac}^{n}}+K_{AexA\_in}\times\frac{k_{AexA\_in}^{n}}{{Aex}^{n}+k_{AexA\_in}^{n}}-d_{A}\times A=0 (Equation S8)$$

The solution of variable A in Equation *S*8 was the intersection of curves corresponding to Equations *S*9 and *S*10:

$$h\left( A \right)=K_{selfA\_ac}\times\frac{A^{n}}{A^{n}+k_{selfA\_ac}^{n}} (Equation S9)$$

$$q\left( A \right)=d_{A}\times A-K_{AexA\_in}\times\frac{k_{AexA\_in}^{n}}{{Aex}^{n}+k_{AexA\_in}^{n}} (Equation S10)$$

In the phase portrait, Equation *S*9 corresponded to black curve, while Equation *S*10 with different parameters (Aex) corresponded to dotted lines in various colors. The black curve had only one intersection point with dotted lines of different colors (Figure S9c). The results revealed that gene A, as described in Equation *S*3, was not bistable.

For the Equation *S*4, when the change rate of gene A over time was equal to zero, Equation *S*4 transformed into Equation *S*11:

$$K_{constitutive}+K_{AexA\_in}\times\frac{k_{AexA\_in}^{n}}{{Aex}^{n}+k_{AexA\_in}^{n}}-d_{A}\times A=0 (Equation S11)$$

The Equation *S*11 shown that when all of parameters' values were given, the value of variable A was uniquely determined (Figure S9e). Therefore, gene A described in Equation *S*4 was not bistable.

The results of the above analysis suggested that if gene A's bi-stability was expected to be realized in a continuous model, gene A's expression over time should be described in the form of Equation *S*2, with intracellular self-activation and intercellular lateral inhibition integrated through AND-gate.

## Ordinary differential equations that characterize the genetic network T1 in the continuous model

For two-gene growing system, the network T1 has four regulatory edges: intracellular self-activation of gene A, intercellular lateral inhibition of genes A and B, and cross-cell activation from gene A to gene B in separate cells (Figure 2c). Genes A and B each contain two regulatory edges, and the regulatory logic between these two edges could be AND or OR in converting the Boolean models to continuous models.

In the two-gene system regulated by the genetic network T1, when the system reached generation 3, the Boolean model predicted that cells with two neighbors would have low gene A expression, while cells with only one neighbor would have high gene A expression. When matching to a single-gene system simulated by the continuous model, the pattern generated by three equations (Equation *S*2-*S*4) all had similar properties (Figure 4a, 4b and 4c). When the system reached generation 4, however, gene A expression levels in the central four cells were low, while gene A expression levels in the four cells on both ends of the system were high. Only Equation *S*2 could reproduce the same expression pattern of gene A in the continuous model (Figure 4a). As a result, Equation *S*2 should be used to describe the changing expression of gene A in the genetic network T1 in the continuous model.

However, the expression pattern obtained from the Boolean model for gene B in the genetic network T1 could not provide information regarding which regulation logic should be utilized for gene B in continuous model. As a result, we used the parameter screening method (100,000 sets of parameters) to find acceptable parameters that could produce the standard pattern in the continuous model, using the pattern formed in generation 4 acquired in the Boolean model as the judgment standard. We designed four regulation logic combinations: gene A and gene B were both OR-gate; gene A was OR-gate while gene B was AND-gate; gene A was AND-gate while gene B was OR-gate; gene A and gene B were both AND-gate. We discovered that there were 10, 29, 4020, and 6953 parameter sets corresponding to these four regulation logic combinations that could produce standard patterns, respectively.

The aforementioned results demonstrated that in the process of transforming the genetic network T1 from Boolean model to continuous model, gene A in the genetic network T1 required to adopt AND-gate, but gene B in the genetic network T1 had no special needs for regulation logic. The Equation *S*2 was used to describe the variation of gene A in the genetic network T1 over time in the continuous model, whereas Equations *S*12 and *S*13 were used to describe the variation of gene B in the genetic network T1 over time under various regulation logic (Figure 4e and S10c):

$$\frac{dB}{dt}=K_{AexB_{ac}}\times\frac{{Aex}^{n}}{{Aex}^{n}+k_{AexB_{ac}}^{n}}+K_{BexB_{in}}\times\frac{k_{BexB_{in}}^{n}}{{Bex}^{n}+k_{BexB_{in}}^{n}}-d_{B}\times B (Equation S12)$$

$$\frac{dB}{dt}=K_{AexB_{ac}}\times\frac{{Aex}^{n}}{{Aex}^{n}+k_{AexB_{ac}}^{n}}\times K_{BexB_{in}}\times\frac{k_{BexB_{in}}^{n}}{{Bex}^{n}+k_{BexB_{in}}^{n}}-d_{B}\times B (Equation S13)$$

## Stochastic differential equations

We introduced white noise to the original deterministic equations representing gene expression variations to investigate the influence of noise on pattern formation and stability. Stochastic differential equations contain drift term and diffusion term. Therefore, we added the diffusion term, $\frac{\lambda}{\surd dt}$ to the original equations to construct stochastic differential equations.

## The derivation from the genetic network T1 to the genetic network T6

We derived the equations describing the changes of genes A and B in the genetic network T1 over time in the continuous model by scanning the parameter space. When simulating one-dimensional division-decision systems with these two sets of equations, we found that systems gain the maximum positional information in generation 4. However, we noticed that, in addition to gene A, gene B was also bistable when we looked at the phase portrait of gene B in the Boolean model. The expression level of gene B was dictated by the expression levels of gene A and gene B in nearby cells in the Boolean model. When the expression levels of genes A and B in nearby cells were used as parameters, we discovered that the expression levels of intracellular gene B were similarly influenced by the initial expression levels of gene B. In the parameter space where the expression levels of gene A and gene B were comparable in nearby cells, gene B looked bistable (Figure S11a and S11b).

In network T1, gene B was regulated by two regulatory edges, which were activated by gene A in nearby cells and inhibited by gene B in nearby cells. At the transition into continuous model section, we found that the expression change of gene B over time could be described by Equations *S*12 or *S*13 under different regulation logics. When the change rate of gene B over time was zero, indicating that gene B's expression level had stabilized, the expression levels of gene A and B in nearby cells and the expression level of gene B in cells exhibited the following relationship:

$$K_{AexB_{ac}}\times\frac{{Aex}^{n}}{{Aex}^{n}+k_{AexB_{ac}}^{n}}+K_{BexB_{in}}\times\frac{k_{BexB_{in}}^{n}}{{Bex}^{n}+k_{BexB_{in}}^{n}}=d_{B}\times B (Equation S14)$$

$$K_{AexB_{ac}}\times\frac{{Aex}^{n}}{{Aex}^{n}+k_{AexB_{ac}}^{n}}\times K_{BexB_{in}}\times\frac{k_{BexB_{in}}^{n}}{{Bex}^{n}+k_{BexB_{in}}^{n}}=d_{B}\times B (Equation S15)$$

Equation *S*14 showed that the stable state value of gene B in cells was modulated when the expression levels of gene A and gene B in nearby cells varied (Figure S10b). If the expression level of gene A in nearby cells was zero, however, a change in gene B expression level in nearby cells had no effect on the stable state value of gene B in the cell in Equation *S*15 (Figure S10e). Despite the fact that Equation *S*12 could guarantee that external activator and inhibitor genes controlled the stable state of intracellular gene B, gene B controlled by Equation *S*12 did not exhibit bi-stability. As a consequence, Equation *S*12 was modified to include self-activation regulation of intracellular gene B, and Equation *S*16 was constructed to quantify gene B's rate of change over time:

$$\frac{dB}{dt}=K_{selfB_{ac}}\times\frac{B^{n}}{B^{n}+k_{selfB_{ac}}^{n}}+K_{AexB_{ac}}\times\frac{{Aex}^{n}}{{Aex}^{n}+k_{AexB_{ac}}^{n}}$$

$$+K_{BexB_{in}}\times\frac{k_{BexB_{in}}^{n}}{{Bex}^{n}+k_{BexB_{in}}^{n}}-d_{B}\times B (Equation S16)$$

Equation *S*16 was altered to Equation *S*17 when the change rate of gene B was equal to zero, and the expression level of the corresponding gene B reached a stable state:

$$K_{selfB_{ac}}\times\frac{B^{n}}{B^{n}+k_{selfB_{ac}}^{n}}={d_{B}\times B-K}_{AexB_{ac}}\times\frac{{Aex}^{n}}{{Aex}^{n}+k_{AexB_{ac}}^{n}}$$

$$-K_{BexB_{in}}\times\frac{k_{BexB_{in}}^{n}}{{Bex}^{n}+k_{BexB_{in}}^{n}} (Equation S17)$$

$$F\left( B \right)=K_{selfB_{ac}}\times\frac{B^{n}}{B^{n}+k_{selfB_{ac}}^{n}} (Equation S18)$$

$$G(B)={d_{B}\times B-K}_{AexB_{ac}}\times\frac{{Aex}^{n}}{{Aex}^{n}+k_{AexB_{ac}}^{n}}-K_{BexB_{in}}\times\frac{k_{BexB_{in}}^{n}}{{Bex}^{n}+k_{BexB_{in}}^{n}} (Equation S19)$$

The left-hand side of the equals sign in Equation *S*17 corresponded to the phase portrait's black curve, while the right-hand side corresponded to the phase portrait's straight line. Fixed points were found at the intersections of a curve and a straight line; stable fixed points were found on the upper and lower sides of the curve; and unstable fixed points were found in the center of the curve. The lack of expression of activator gene A and inhibitor gene B in nearby cells was shown by the black line in the phase portrait. The dark blue and light blue dotted lines represented no expression of gene B in nearby cells, but gradually increased expression of gene A. The dark green and light green dotted lines represented no expression of gene A in nearby cells, but gradually increased expression of gene B. Increased expression of both gene A and gene B in nearby cells was shown by the dark red and light red dotted lines. The stable fixed point moved when the dotted line shifted in the phase portrait, implying that gene A and B expression levels in nearby cells had an influence on the intracellular gene B's stable state and gene B was bistable at the same time (Figure S11c).

The corresponding genetic network was T6 after adding intracellular self-activation of gene B. We applied Equations *S*2 and *S*16 to simulate a one-dimensional division-decision growing system regulated by the genetic network T6, and discovered that in generation 4, four distinct cell states formed, and the system's positional information reached its maximum value (Figure S11d).

## Parameter values in the continuous model

In simulating the temporal dynamics of gene expression within single-gene systems, we utilized the continuous model Equations *S*2, *S*3, and *S*4, which contain five, five, and four parameters, respectively. We specified the parameter sets for each equation corresponding to the conditions illustrated in Figures 4a, 4b, and 4c, with these sets enumerated in Table A (In all equations, the value of n is uniformly set to 2).

To explore how the parameters influenced the formation of multicellular stable states with maximum positional information (1.0 bit) at the 8-cell stage in single-gene systems, we conducted a parameter sensitivity analysis. We systematically varied each parameter to establish its permissible range that still allowed for the stable state to manifest. Beginning with the baseline parameter values, we increased each by a percentage increment, continuing until the system no longer attained the desired stable state with maximum positional information. This percentage defined the upper limit for parameter variation, while the lower limit was ascertained similarly. Permissible parameter variations for Equations *S*2, *S*3, and *S*4 are presented in Table A.

The data in Table A demonstrate a marked discrepancy in parameter variation between Equation *S*2 and Equations *S*3 and *S*4. This distinction corroborates our prior stability analysis: the depiction in Figure 4a, governed by Equation *S*2, requires the maintenance of gene A's bi-stability. Hence, the parameter selection for Equation *S*2 is tightly regulated to ensure the bistability of gene A is preserved. The admissible parameter range for Equation *S*2 is therefore significantly narrower compared to those for Equations *S*3 and *S*4.

Table A. Values of parameters in the continuous model of the single-gene systems and the upper/lower limits of parameter variability.

| Equation S2 (AND-gate) | | | | | |
| --- | --- | --- | --- | --- | --- |
| Parameter | K_selfA_ac_ | k_selfA_ac_ | K_AexA_in_ | k_AexA_in_ | d_A_ |
| Value | 0.14981 | 0.2106 | 1.1816 | 0.752 | 0.1695 |
| Upper Limit of Parameter Increase | 30% | 20% | 30% | 20% | 120% |
| Lower Limit of Parameter Decrease | 50% | 20% | 50% | 10% | 30% |
| Equation S3 (OR-gate) | | | | | |
| Parameter | K_selfA_ac_ | k_selfA_ac_ | K_AexA_in_ | k_AexA_in_ | d_A_ |
| Value | 0.1 | 1.8 | 6.564 | 0.0517 | 6.5867 |
| Upper Limit of Parameter Increase | >200% | >200% | >200% | >200% | >200% |
| Lower Limit of Parameter Decrease | >99% | >99% | 90% | >99% | >99% |
| Equation S4 (constitutive) | | | | | |
| Parameter | K_constitutive_ | | K_AexA_in_ | k_AexA_in_ | d_A_ |
| Value | 0.035 | | 3.406 | 0.1587 | 3.4036 |
| Upper Limit of Parameter Increase | >200% | | >200% | >200% | >200% |
| Lower Limit of Parameter Decrease | >99% | | 70% | 80% | 80% |

For the continuous model simulations of the two-gene systems, we employed Equations *S*2 and *S*12 to generate the results depicted in Figure 4e, Equations *S*2 and *S*13 for Figure S10c, and Equations *S*2 and *S*16 for Figure S11d. The precise parameter values utilized in these simulations are meticulously documented in Table B. Mirroring the approach taken with the single-gene systems, a parameter sensitivity analysis was performed for the equations governing the two-gene systems. The permissible ranges of parameter variation ascertained from this analysis are comprehensively listed in Table B.

Table B. Values of parameters in the continuous model of the two-gene systems and the upper/lower limits of parameter variability.

| Equation S2 (AND-gate)  Equation S12 (OR -gate) | | | | | | | |
| --- | --- | --- | --- | --- | --- | --- | --- |
| Parameter | K_selfA_ac_ | k_selfA_ac_ | K_AexA_in_ | k_AexA_in_ | d_A_ |  |  |
| Value | 0.0169 | 0.1547 | 4.2664 | 0.3352 | 0.0702 |  |  |
| Upper Limit of Parameter Increase | >200% | 110% | >200% | 110% | 180% |  |  |
| Lower Limit of Parameter Decrease | 96% | 60% | 60% | 80% | 94% |  |  |
| Parameter | K_AexBac_ | k_AexBac_ | K_BexBin_ | k_BexBin_ | d_B_ |  |  |
| Value | 0.021 | 2.663 | 0.3219 | 0.0409 | 0.3218 |  |  |
| Upper Limit of Parameter Increase | >200% | >200% | >200% | >200% | >200% |  |  |
| Lower Limit of Parameter Decrease | >99% | >99% | 94% | >99% | 80% |  |  |
| Equation S2 (AND-gate)  Equation S13 (AND -gate) | | | | | | | |
| Parameter | K_selfA_ac_ | k_selfA_ac_ | K_AexA_in_ | k_AexA_in_ | d_A_ |  |  |
| Value | 0.144 | 0.09 | 0.7717 | 0.3088 | 0.1103 |  |  |
| Upper Limit of Parameter Increase | >200% | >200% | >200% | 50% | >200% |  |  |
| Lower Limit of Parameter Decrease | 70% | 50% | 70% | 70% | 95% |  |  |
| Parameter | K_AexBac_ | k_AexBac_ | K_BexBin_ | k_BexBin_ | d_B_ |  |  |
| Value | 0.1972 | 0.7081 | 2.179 | 0.1156 | 0.2862 |  |  |
| Upper Limit of Parameter Increase | >200% | 140% | >200% | >200% | >200% |  |  |
| Lower Limit of Parameter Decrease | 80% | >99% | 80% | >99% | 80% |  |  |
| Equation S2 (AND-gate)  Equation S16 (OR-gate) | | | | | | | |
| Parameter | K_selfA_ac_ | k_selfA_ac_ | K_AexA_in_ | k_AexA_in_ | d_A_ |  |  |
| Value | 0.144 | 0.09 | 0.7717 | 0.3088 | 0.1103 |  |  |
| Upper Limit of Parameter Increase | 170% | >200% | 170% | 50% | >200% |  |  |
| Lower Limit of Parameter Decrease | 80% | 50% | 80% | 94% | 60% |  |  |
| Parameter | K_selfBac_ | k_selfBac_ | K_AexBac_ | k_AexBac_ | K_BexBin_ | k_BexBin_ | d_B_ |
| Value | 0.0525 | 3.3223 | 0.1 | 0.7081 | 0.2165 | 0.1156 | 0.2862 |
| Upper Limit of Parameter Increase | >200% | >200% | 80% | >200% | >200% | >200% | >200% |
| Lower Limit of Parameter Decrease | >99% | 94% | >99% | 30% | 60% | 60% | 60% |

# The booleanization of gene expression profiles and the identification of the cell state in *C.elegans* early development

A total of 195 measurements of the *C.elegans* early development were obtained from the EPIC database (<http://epic.gs.washington.edu/>) [18, 52]. Each measurement traces a single worm from the zygotic stage to around 300 cell stage, including the mCherry-labeled transcription of a developmentally important gene, and the GFP-labeled nucleus location. The 195 embryos trace the time courses of 112 developmentally important transcription factors, with some factors measured more than once. To represent the cell state transition events occurring before time 140, we selected 40 lineage-specific genes and binarized them. A lineage specifier is defined as following. When cell $C$ divides into $C1$ and $C2$, $C1$ and $C2$ form two descendant lineages. The expression levels of gene $g$ in these two daughter lineages at time *t* are scored by Student’s t-test to evaluate how well this gene can separate these two daughter lineages. If the max score is larger than a threshold, we define gene $g$ as a lineage specifier for division $C$. For example, in Figure S12a, gene TBX-38 is a lineage specifier for the division of AB. The values of this gene in two descendant lineages are then binarized to one (in the lineage with higher expression) and zero (in the lineage with lower expression). The distance between cells on the binarized gene-expression space of these 40 genes positively correlated to the distance on the continuous gene expression space for all 112 genes (Figure S12b, S12c and S12d). All 371 cells have 40 binarized genes, yielding 78 distinct Boolean profiles that correspond to 78 states (77 state transition events). We named each state by the lower-case name of the first cell entering this state. For example, cells P1, P2, P3, P4 all have the same gene expression profile, and their states are all “p1”.
