# Supplementary material for: A mathematical framework for understanding the spontaneous emergence of complexity applicable to growing multicellular systems
Source: PLoS Comput Biol. 2024 Jun 5;20(6):e1011882. doi: 10.1371/journal.pcbi.1011882 (PMC11182560; doi:10.1371/journal.pcbi.1011882)
Supplement: S5 Appendix — (ZIP) [file pcbi.1011882.s005.zip › PositionalInformation_Code/README.docx]

This README file contains instructions for the simulation code used in the “A mathematical framework for understanding the spontaneous emergence of complexity applicable to growing multicellular systems”. The code is organized into two main parts to simulate different aspects of positional information. Below is a detailed explanation of which scripts correspond to which parts of the paper and how to generate specific figures.

Folder Structure:
The ‘PositionalInformation_Code’ folder contains two subfolders:

- ‘Boolean_model’: This folder contains the programs associated with the Boolean model part of the paper.
- ‘Continues_model’: This folder contains the programs related to the continuous model part of the paper.

Each of these subfolders contains further subdivisions for the ‘Single_gene_system’ and ‘Two_gene_system’ simulations.

To simulate the system's development from a single cell to a multicellular state and generate the corresponding data, run the following MATLAB files:

1. ‘cell_farm_1D.m’ simulates the development of the system from a single cell into a multicellular state. This simulation applies to both Boolean and continuous models and both single and two-gene systems, depending on the folder it is located in.
2. ‘cell_farm_screen.m’ simulates screening for genetic networks that meet specific conditions.
3. ‘cell_farm_noise.m’ simulates the system's development while accounting for noise in gene expression updates.
4. ‘cell_farm_asynchronous.m’ simulates the system's development with the possibility of cells undergoing asynchronous division.
5. ‘cell_farm_nodivi_reachstablestate.m’ simulates the development of a multicellular system with a fixed number of cells over time.
6. ‘cell_farm_1D_fixedone.m’ simulates the development of the system from two cells to a multicellular state with one end of the cell chain fixed and non-dividing.

These files should be run within their respective subdirectories, depending on whether you are working with a single or two-gene system and whether you are examining the Boolean or continuous model.

Figure Generation:

1. ‘PositionalInformation_Code/Boolean_model/Single_gene_system/cell_farm_1D.m’ generates data for Figure 1e.
2. ‘PositionalInformation_Code/Boolean_model/Two_gene_system/cell_farm/cell_farm_1D.m’ generates data for Figure 2d.
3. ‘PositionalInformation_Code/Boolean_model/Two_gene_system/Topology_Screen/screen_topology_initial_A1_B0/draw_PI_motif_majorfigure.m’ generates Figure 2g.
4. ‘PositionalInformation_Code/Boolean_model/Two_gene_system/Fixed_Cell_Number/Cell_8/8cells_basin_size_stable_states_number.fig’ generates Figure 3b.
5. ‘PositionalInformation_Code/Boolean_model/Two_gene_system/Fixed_Cell_Number/Cell_4/draw_basinsize_bar.m’ generates Figure 3c.
6. ‘PositionalInformation_Code/Boolean_model/Two_gene_system/Fixed_Cell_Number/Cell_8/draw_basinsize.m’ generates Figure 3d.
7. ‘PositionalInformation_Code/Boolean_model/Two_gene_system/Update_noise/draw_percent.m’ generates Figure 3f.
8. ‘PositionalInformation_Code/Boolean_model/Two_gene_system/Asynchronous/draw_asynchronous.m’ generates Figure 3g.
9. ‘PositionalInformation_Code/Continues_model/Single_gene_system/AND-gate/cell_farm_1D.m’ generates Figure 4a.
10. ‘PositionalInformation_Code/Continues_model/Single_gene_system/OR-gate/SelfactiveA/cell_farm_1D.m’ generates Figure 4b.
11. ‘PositionalInformation_Code/Continues_model/Single_gene_system/OR-gate/ConstitutiveA/cell_farm_1D.m’ generates Figure 4c.
12. ‘PositionalInformation_Code/Continues_model/Single_gene_system/Noise/Generation3/draw_noise.m’ generates Figure 4d.
13. ‘PositionalInformation_Code/Continues_model/Two_gene_system/T1/AB_plus_add/cell_farm_1D.m’ generates Figure 4e.
14. ‘PositionalInformation_Code/Continues_model/Two_gene_system/Noise/draw_noise.m’ generates Figure 4f.
15. ‘PositionalInformation_Code/Continues_model/Single_gene_system/AND-gate/Aex_A_phase_diagram.m’ generates Figure S9b.
16. ‘PositionalInformation_Code/Continues_model/Single_gene_system/OR-gate/SelfactiveA/Aex_A_phase_diagram.m’ generates Figure S9d.
17. ‘PositionalInformation_Code/Continues_model/Single_gene_system/OR-gate/ConstitutiveA/Aex_A_phase_diagram.m’ generates Figure S9e.
18. ‘PositionalInformation_Code/Continues_model/Single_gene_system/Noise/Generation4/draw_noise.m’ generates Figure S9f.
19. ‘PositionalInformation_Code/Continues_model/Two_gene_system/T1/AB_plus_plus/cell_farm_1D.m’ generates Figure S10c.
20. ‘PositionalInformation_Code/Continues_model/Two_gene_system/T6/cell_farm_1D.m’ generates Figure S11d.

Instructions for use:

1. Ensure MATLAB is installed and properly set up on your computer.
2. Navigate to the directory containing the desired script.
3. Run the MATLAB script that corresponds to the figure you want to generate.

Example:
To generate Figure 1e, execute the following in MATLAB:

cd …/PositionalInformation_Code/Boolean_model/Single_gene_system

run cell_farm_1D
