# Supplementary figures and images for: A mathematical framework for understanding the spontaneous emergence of complexity applicable to growing multicellular systems
Source: PLoS Comput Biol. 2024 Jun 5;20(6):e1011882. doi: 10.1371/journal.pcbi.1011882 (PMC11182560; doi:10.1371/journal.pcbi.1011882)

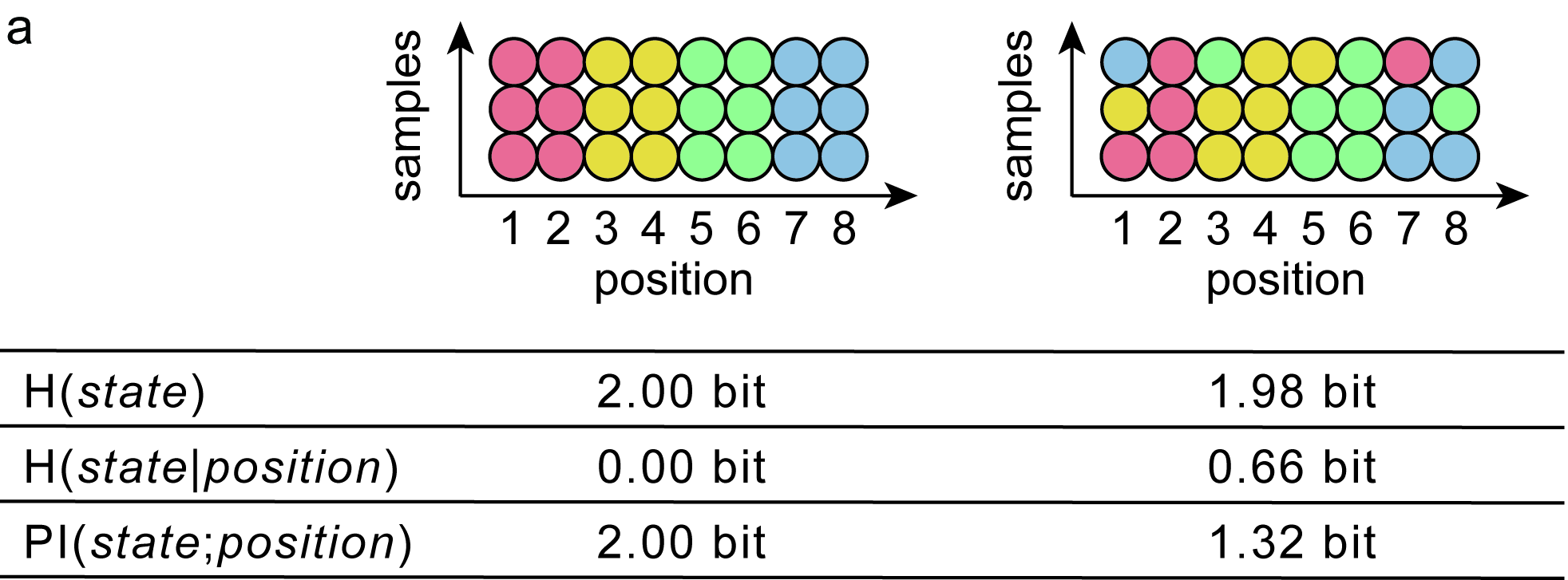

Supplement: S1 Fig — (a). Shannon entropy of cell states minus conditional entropy of cell states equals positional information. The conditional entropy is low (left table) if the cell state (cell states are displayed in different colors) is defined according to the position in several samples; if the cell state is still uncertain when the position is given, the conditional entropy is large (right table). (TIF) [file pcbi.1011882.s006.tif]

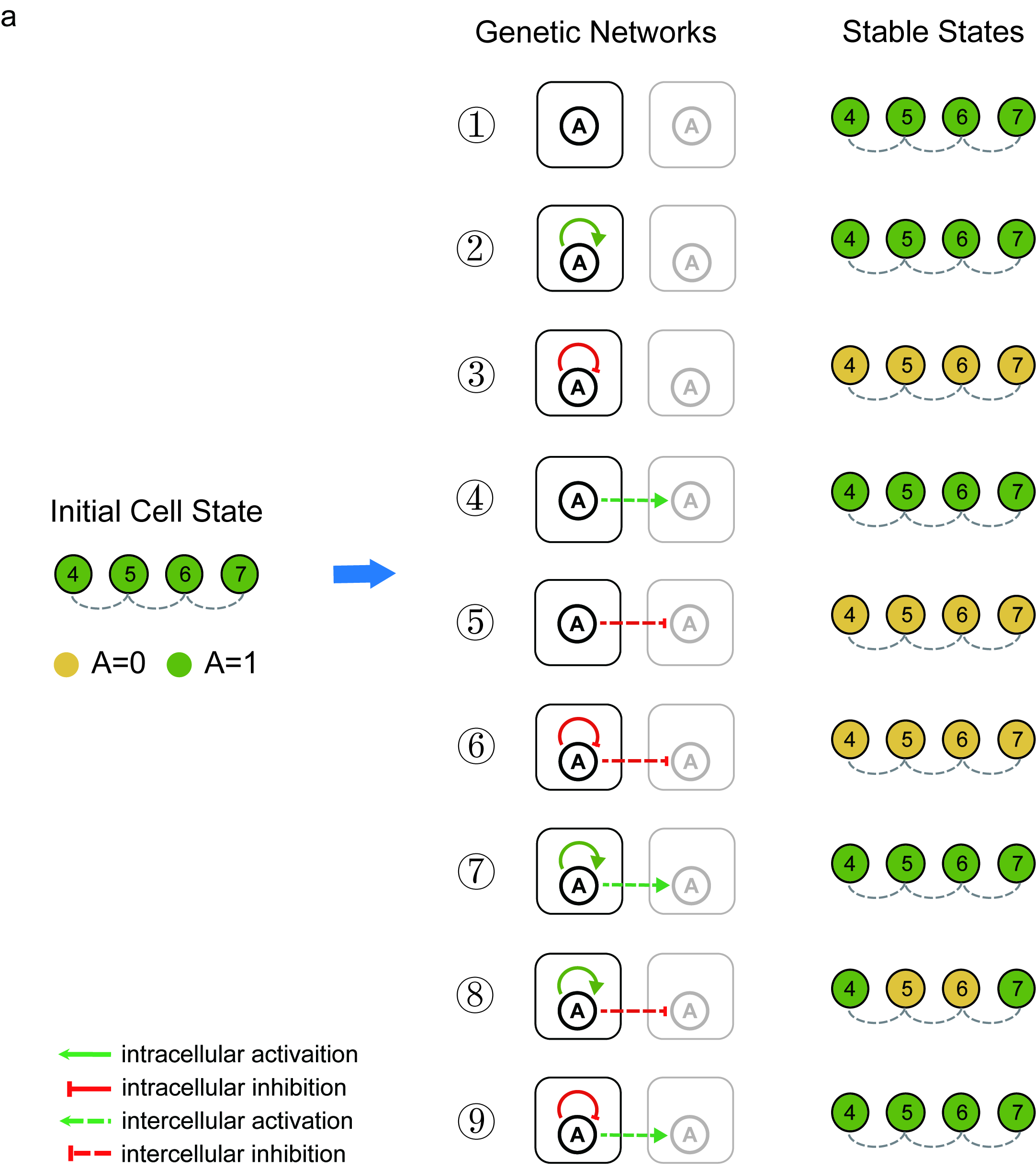

Supplement: S2 Fig — (a). Starting with high levels of gene A expression in neighboring four cells, systems can achieve numerous multicellular stable states under the regulation of different genetic networks when the number of cells in the system is kept constant at four (TIF) [file pcbi.1011882.s007.tif]

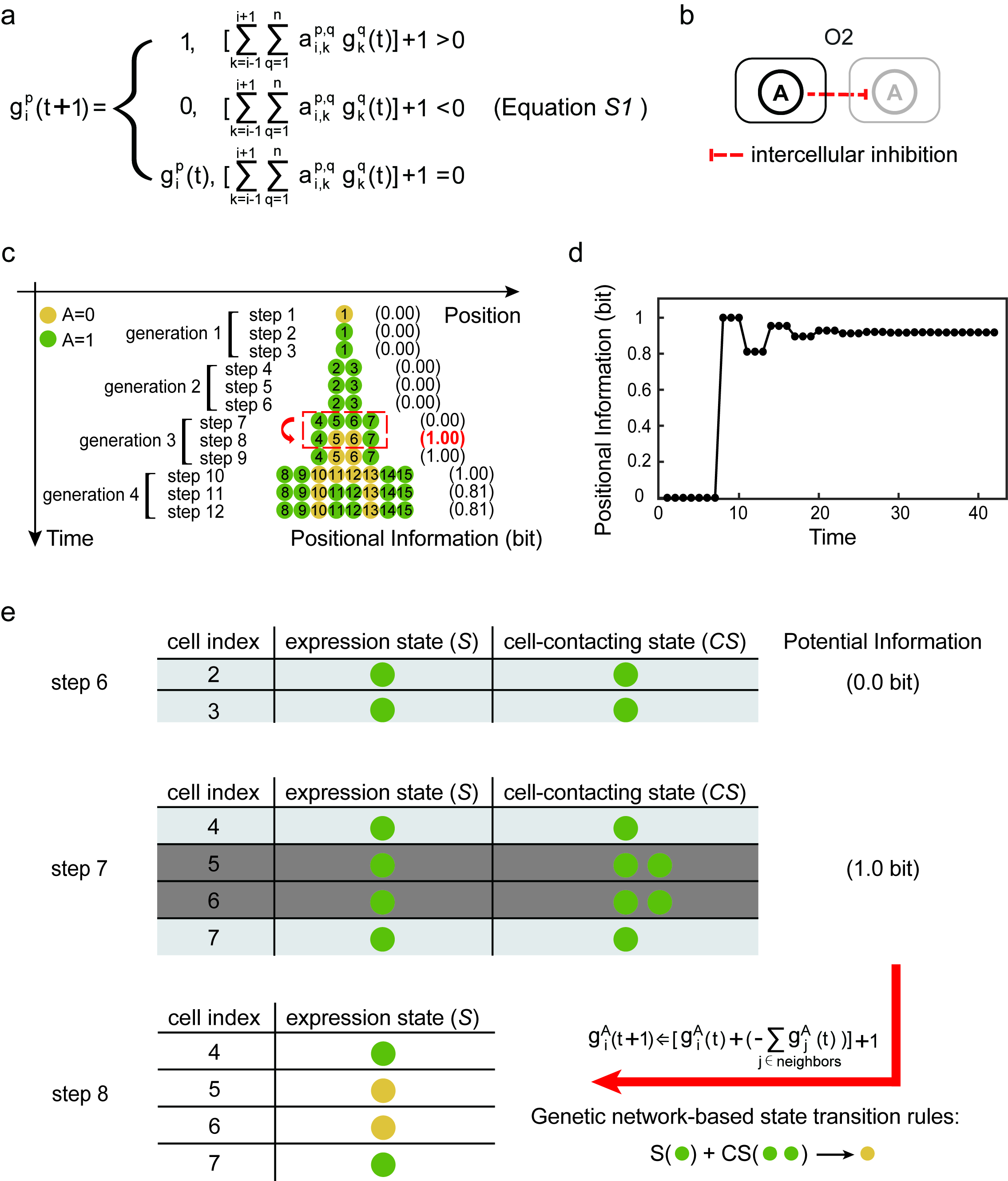

Supplement: S3 Fig — (a). Modified gene expression update function. This modified function determines the gene expression state in the next time step based on the sum of all associated gene expression states in the current time step plus one. (b). The genetic network which controls maximum positional information pattern formation in the single-gene system. (c). The pattern of gene expression varies over time in a single-gene system regulated by the genetic network O2. At step 8, the system obtains 1.0 bit positional information. The time-course of positional information is calculated and shown in the right of the pattern. The binarized gene A expression level determines the cell state. Green circles represent gene A expressed while yellow circles represent gene A unexpressed. (d). The relationship between positional information and time in the growing system regulated by the genetic network O2. (e). Mechanisms of increased positional information in the single-gene system. The maximum positional information 1.0 bit is created by the new space network resulted from cell division. Cell 4, cell 5, cell 6, and cell 7 inherit cell states from their mothers when cell 2 and cell 3 divide. The expression state matrix (S) and the cell-contacting state matrix (CS) comprised the concatenated matrix ([S,C∙S]), which changed after cell division. The new concatenated matrix contains 1.0 bit potential information which could potentially distinguish different two cell states. With the time moving forward, updated cell states according to the genetic network O2 in (b), cell 5 and cell 6 acquires new cell states. At same time, positional information in 4-cell stage increases to 1.0 bit. (TIF) [file pcbi.1011882.s008.tif]

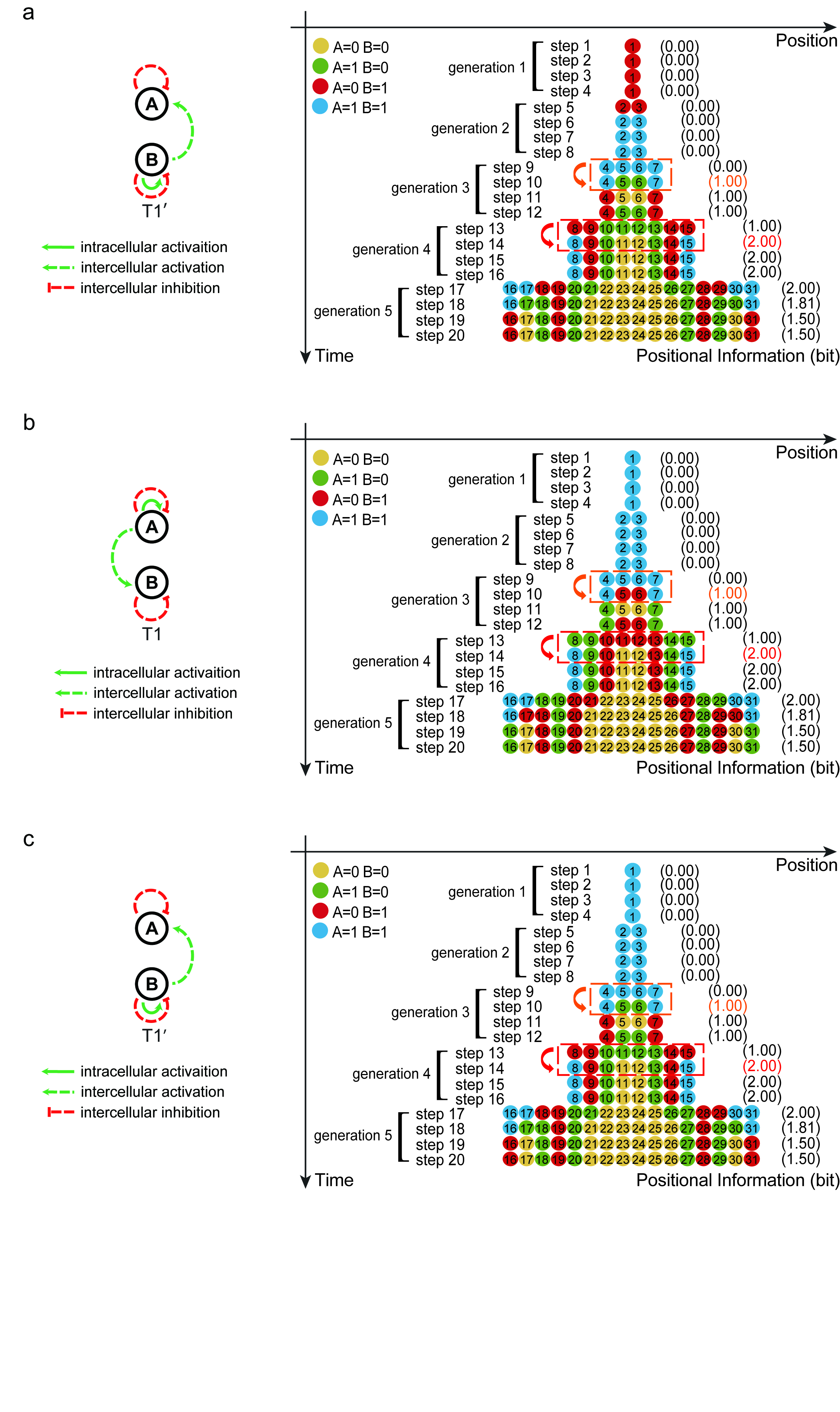

Supplement: S4 Fig — (a). Genetic network T1’ in the two-gene system that regulates maximum positional information pattern formation in a stable state starting with the cell state G→1tinitial=g1Atinitial=0,g1Btinitial=1. At step 14, system reaches 2.0 bit positional information. (b). Genetic network T1 in the two-gene system that regulates maximum positional information pattern formation in a stable state starting with the cell state G→1tinitial=g1Atinitial=1,g1Btinitial=1. At step 14, system reaches 2.0 bit positional information. (c). Genetic network T1’ in the two-gene system that regulates maximum positional information pattern formation in a stable state starting with the cell state G→1tinitial=g1Atinitial=1,g1Btinitial=1. At step 14, system reaches 2.0 bit positional information. (TIF) [file pcbi.1011882.s009.tif]

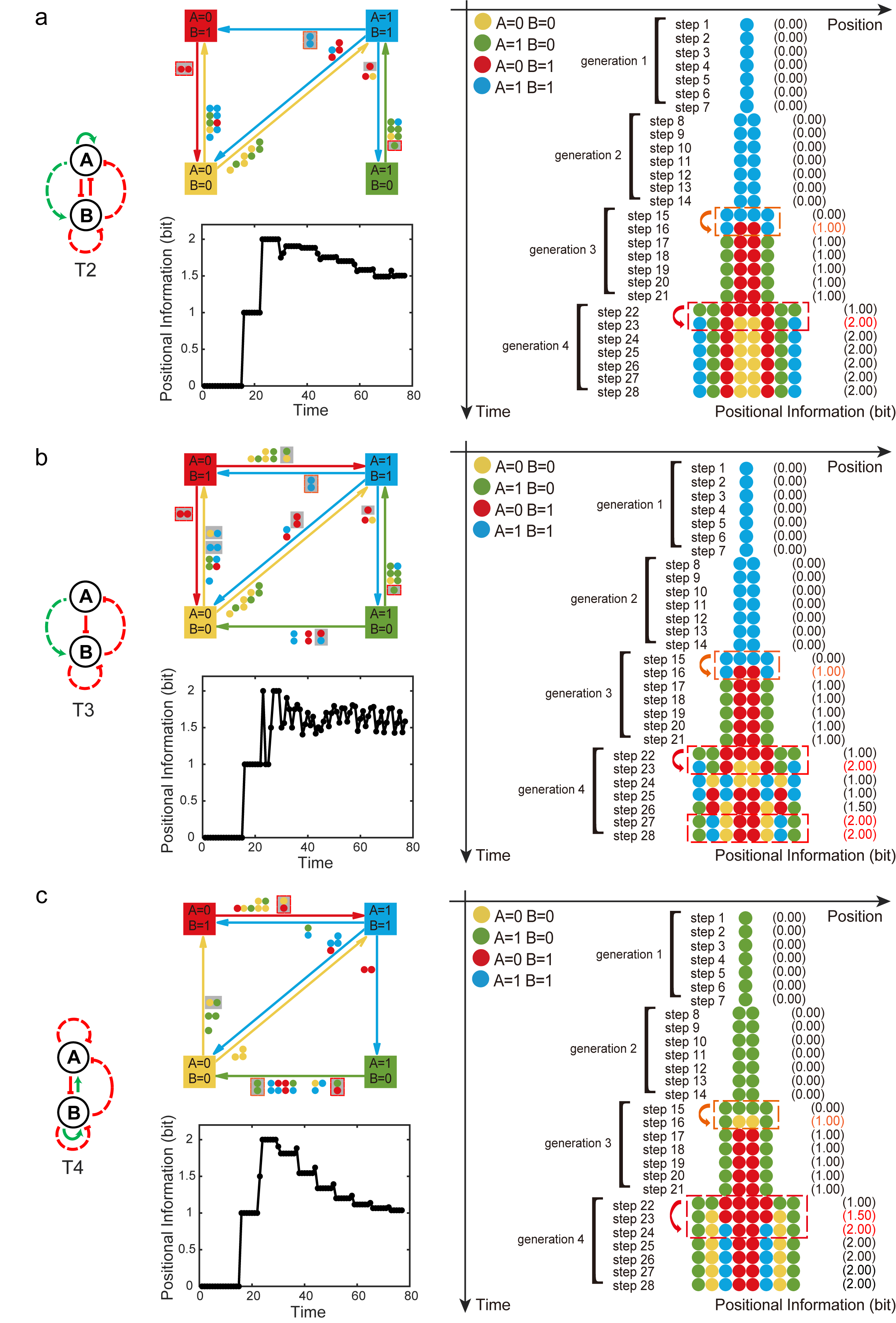

Supplement: S5 Fig — The diagram of transition linkages among four cell states, the relationship between positional information and time, and the time-space pattern of gene expression are depicted under the regulation of the genetic network T2 (a), T3 (b) and T4 (c), respectively. (TIF) [file pcbi.1011882.s010.tif]

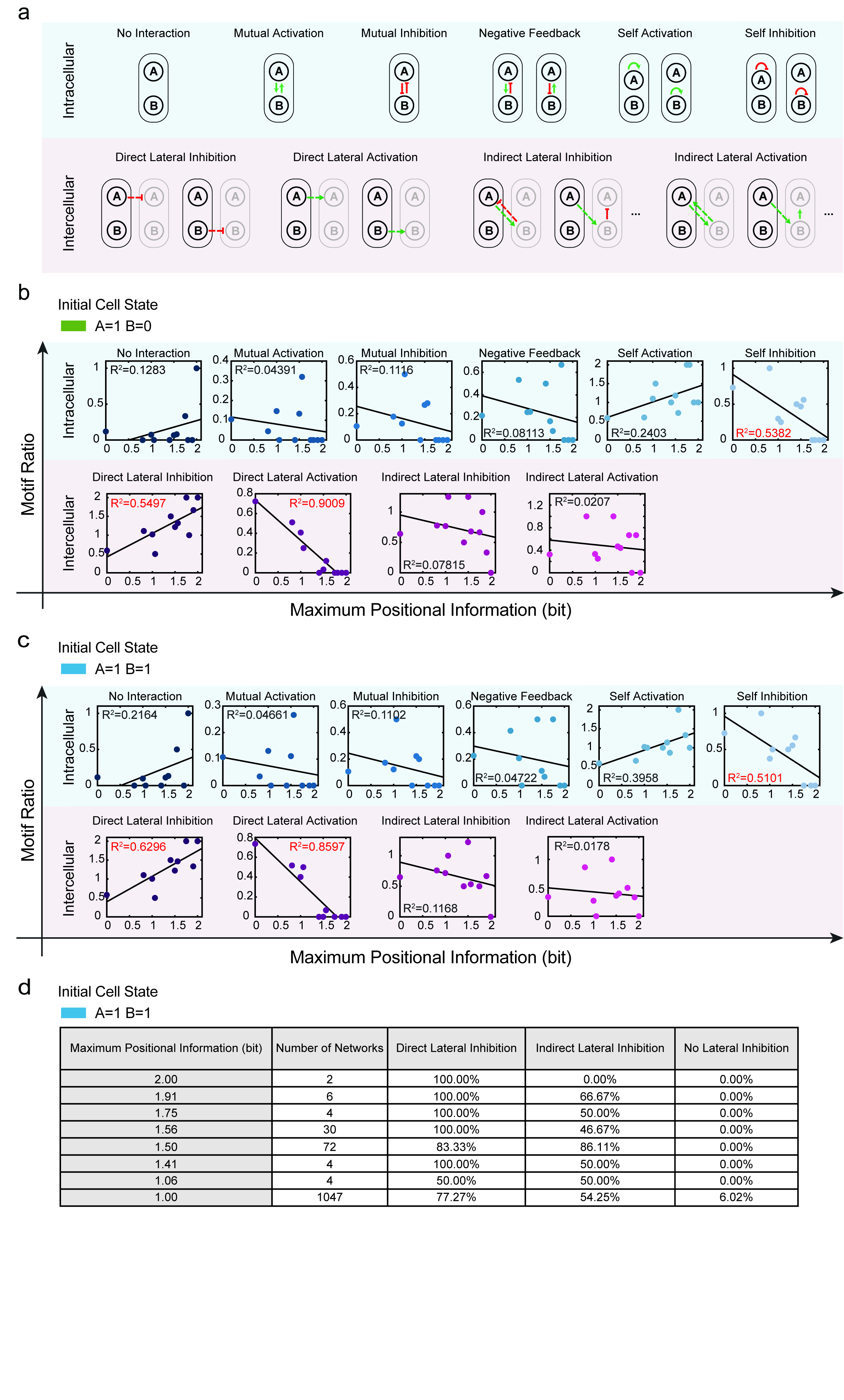

Supplement: S6 Fig — (a). Schematic diagram of intracellular and intercellular regulatory motifs. (b). With the initial cell state: g1Atstart=1,g1Btstart=0, relationships between maximal positional information and the proportion of times the corresponding regulatory motif occurs. The proportion of intracellular self-inhibition and intercellular direct-lateral-activation motifs occurrence are negatively correlated with the maximum positional information. While the proportion of intercellular direct-lateral-inhibition motifs occurrence is positively connected with the maximum positional information. (c). With the initial cell state: g1Atstart=1,g1Btstart=1, relationships between maximal positional information and the proportion of times the corresponding regulatory motif occurs. The proportion of intracellular self-inhibition and intercellular direct-lateral-activation motifs occurrence are negatively correlated with the maximum positional information. While the proportion of intercellular direct-lateral-inhibition motif occurrence is positively connected with the maximum positional information. (d). With the initial cell state: g1Atstart=1,g1Btstart=1, the proportion of occurrence of direct lateral inhibition, indirect lateral inhibition, and no lateral inhibition under various higher than 1.0 bit maximum positional information. (TIF) [file pcbi.1011882.s011.tif]

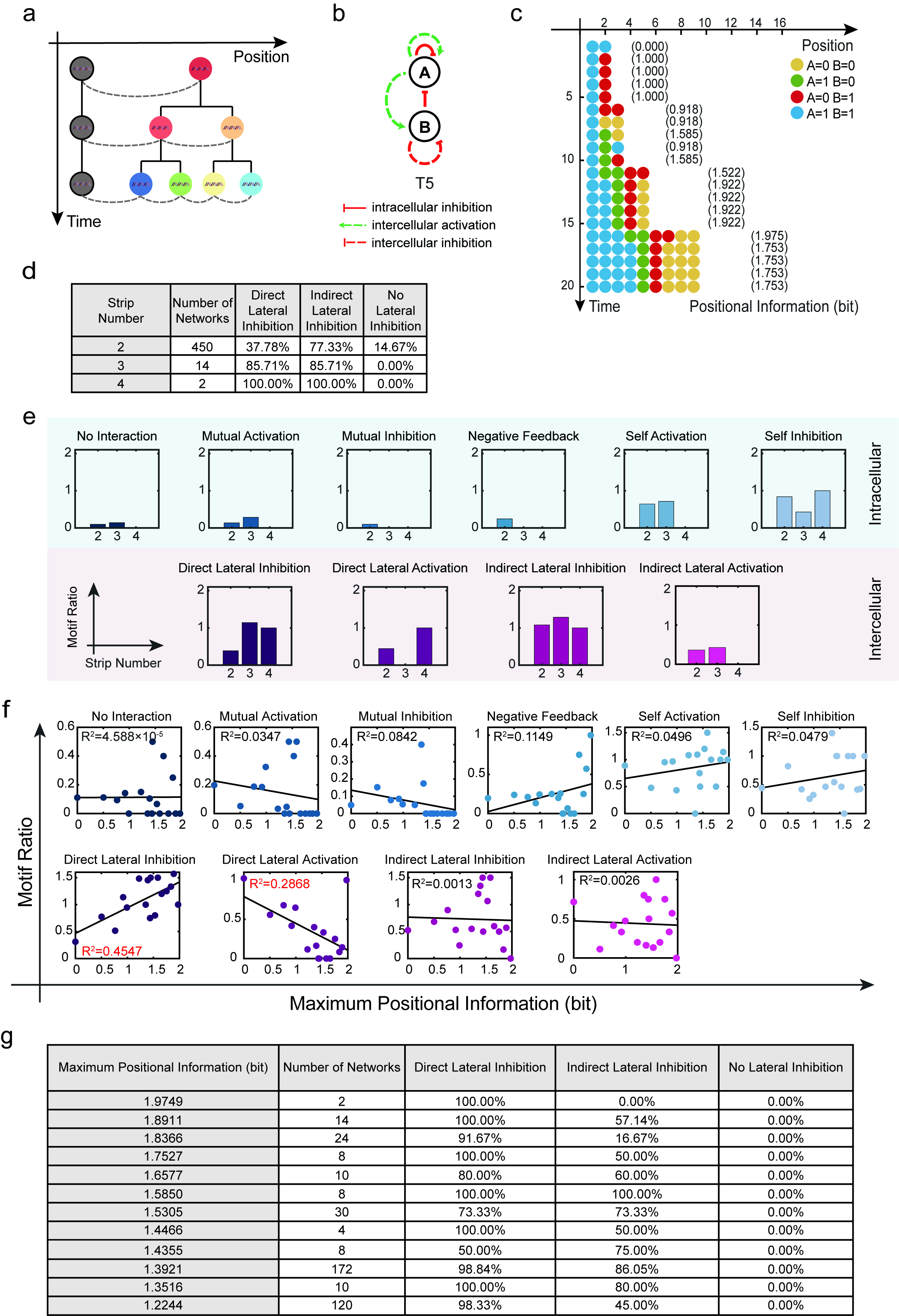

Supplement: S7 Fig — (a). The modified model with a fixed special cell is depicted in this diagram. The cell that is fixed at one end does not divide, and the state of the cell remains the same. With the exception of the fixed cell, all other cells have the ability to divide and update their states. Various colors are used to indicate different cell states. (b). An example of the genetic network (T5) that regulates four blocks pattern formation at the 9-cell stage. (c). The time-space pattern of gene expression that is regulated by the genetic network T5 in (b). The system divides four discrete blocks with various cell states at the 9-cell stable state. (d). With the initial cell state: g1Atstart=1,g1Btstart=1. and fixed cell state: gfixedA(t)=1,gfixedB(t)=1, the proportion of occurrence of direct lateral inhibition, indirect lateral inhibition, and no lateral inhibition under various block numbers. (e). The distribution of motif ratios associated with different block numbers with the initial cell state: g1Atstart=1,g1Btstart=1 and fixed cell state: gfixedA(t)=1,gfixedB(t)=1. (f). Relationships between maximal positional information and the proportion of times the corresponding regulatory motif occurs with the initial cell state: g1Atstart=1,g1Btstart=1 and fixed cell state: gfixedA(t)=1,gfixedB(t)=1. The proportion of intercellular direct-lateral-activation motif occurrence is negatively correlated with the maximum positional information. While the proportion of intercellular direct-lateral-inhibition motif occurrence is positively connected with the maximum positional information. (g). The fraction of occurrence of direct lateral inhibition, indirect lateral inhibition, and no lateral inhibition under varied higher than 1.0 bit maximum positional information with the initial cell state: g1Atstart=1,g1Btstart=1 and fixed cell state: gfixedA(t)=1,gfixedB(t)=1. (TIF) [file pcbi.1011882.s012.tif]

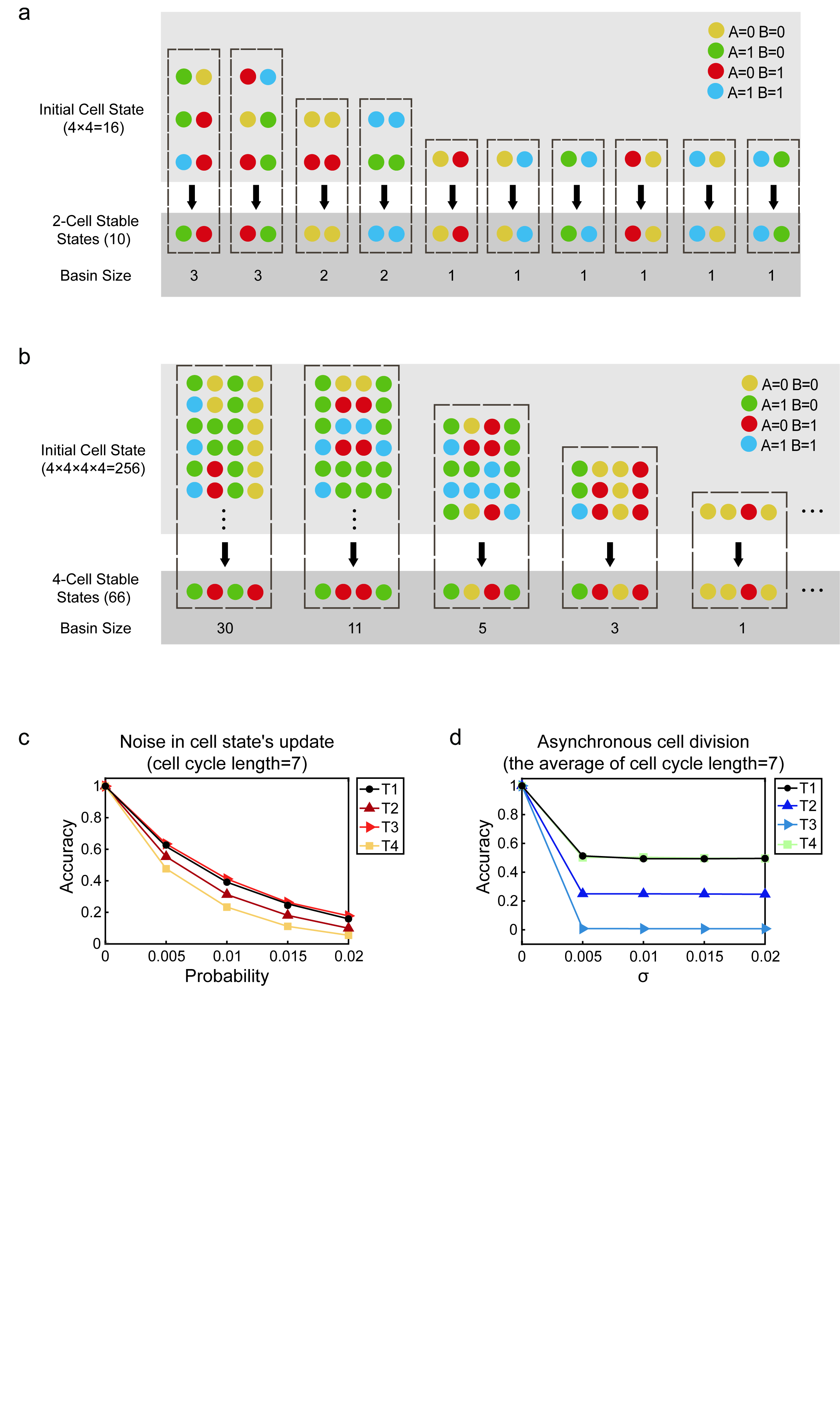

Supplement: S8 Fig — (a). When there are only two cells in the system, there are 16 possible initial multicellular states, but only 10 are stable under the regulation of the genetic network T1. The basin size for a stable state is the number of initial multicellular states that converge to it. (b). In a fixed system with four cells, there are 256 possible initial multicellular states, however only 66 of these multicellular states are stable under the regulation of the genetic network T1. (c). When cell cycle length equals seven, the relationship between the probability of cell state update mistakes and pattern accuracy under the regulation of different genetic networks. (d). When the average cell cycle length equals seven, the relationship between the standard deviation (σ) of cell cycle length and pattern accuracy under the regulation of different genetic networks. (TIF) [file pcbi.1011882.s013.tif]

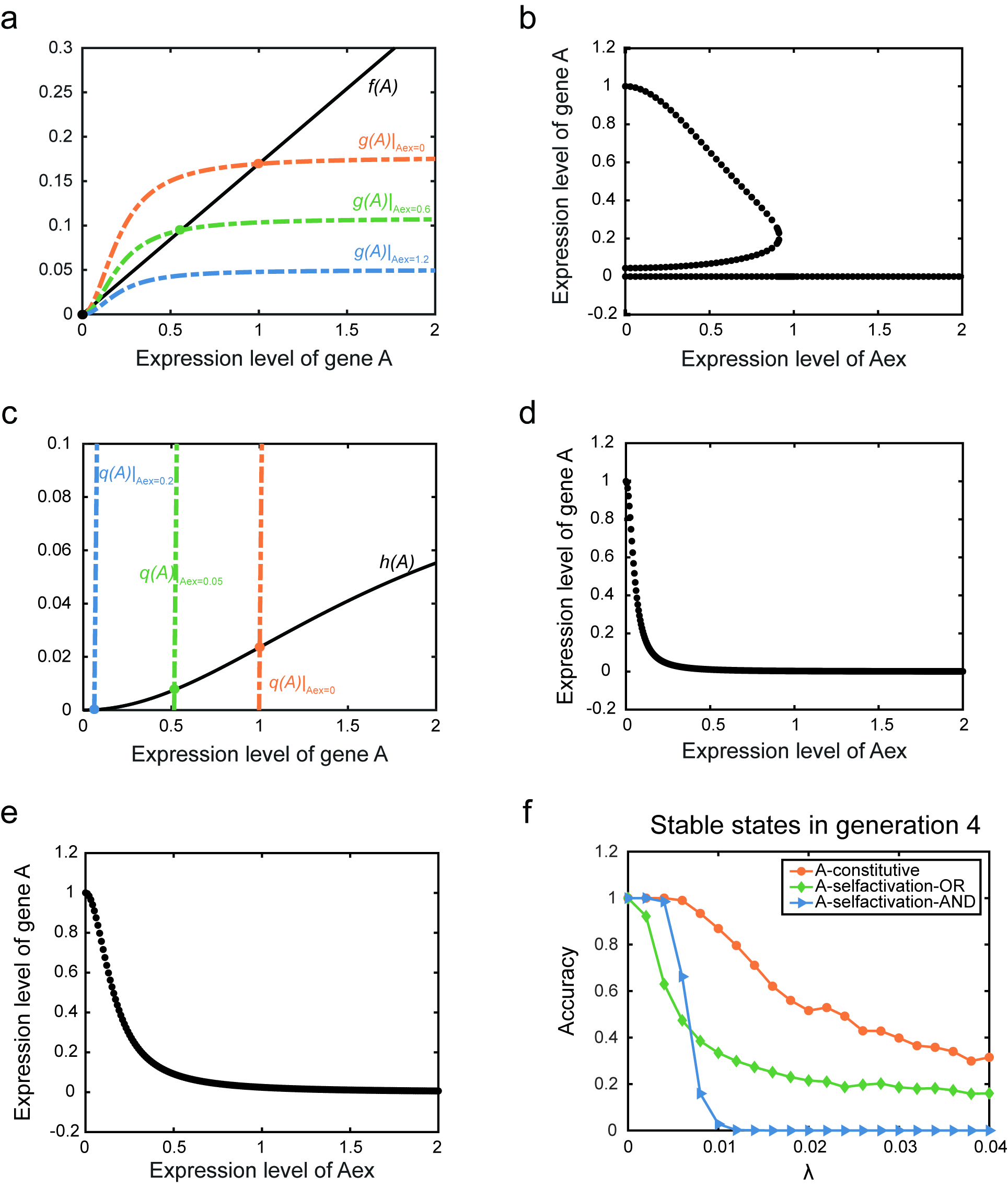

Supplement: S9 Fig — (a). The phase portrait of gene A corresponding to Eq S2. Colored dashed lines represent zero-solution lines for various external parameters. (b). When the expression of gene A is controlled by Eq S2, the relationship between gene A’s fixed points and the expression level of gene A in neighboring cells. (c). The phase portrait of gene A corresponding to Eq S3. Colored dashed lines represent zero-solution lines for various external parameters. (d). When the expression of gene A is controlled by Eq S3, the relationship between gene A’s stable states and the expression level of gene A in neighboring cells. (e). When the expression of gene A is controlled by Eq S4, the relationship between gene A’s stable states and the expression level of gene A in neighboring cells. (f). Resistance to noise of patterns generated in generation 4 under various regulatory settings in single-gene systems. (TIF) [file pcbi.1011882.s014.tif]

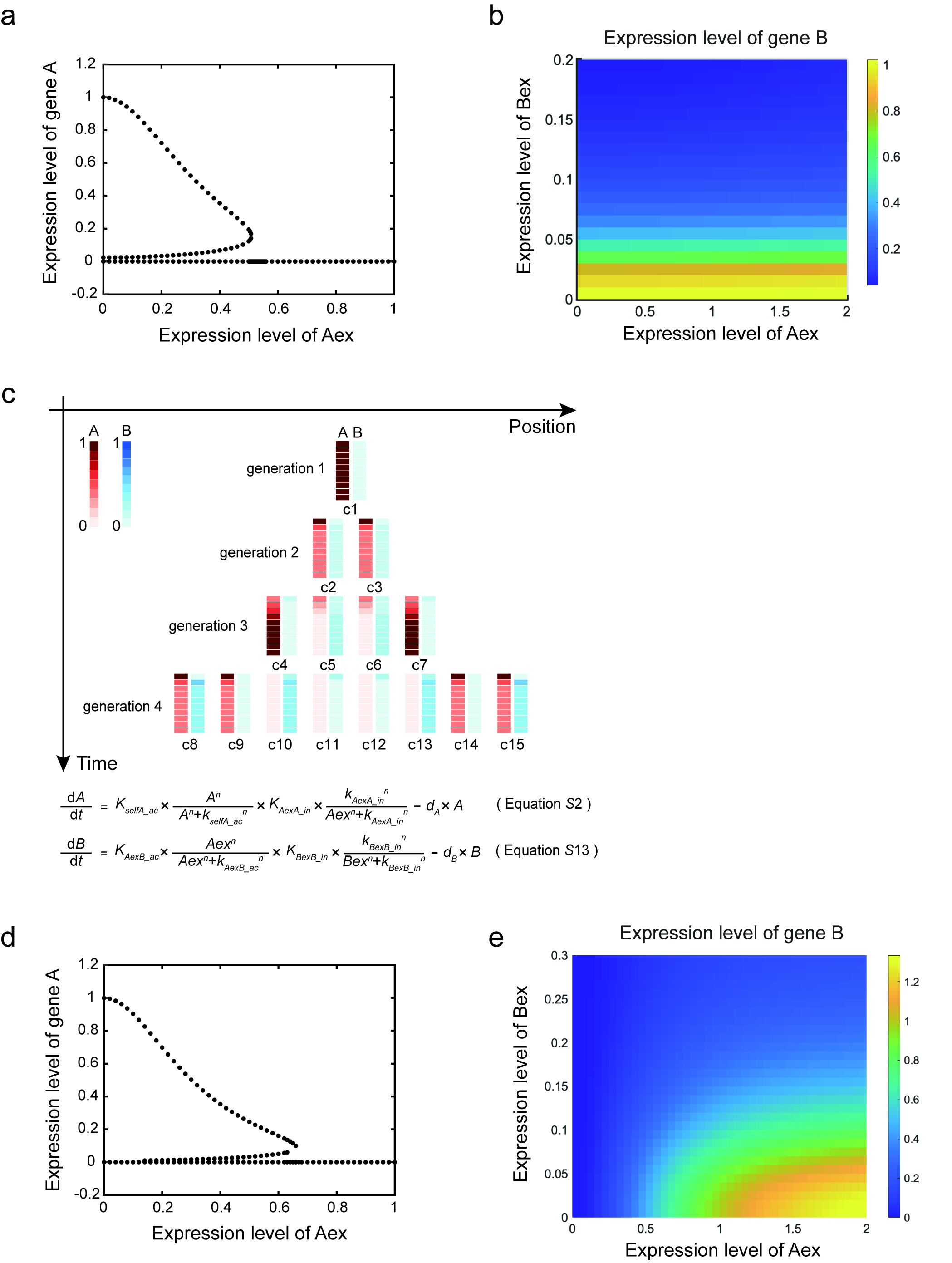

Supplement: S10 Fig — (a). When the expression of gene A in the genetic network T1 is described by Eq S2, the relationship between gene A’s stable states and the expression level of gene A in neighboring cells. (b). When the expression of gene B in the genetic network T1 is described by Eq S12, the intracellular gene B’s stable state value corresponds to two genes in neighboring cells with different expressions. (c). The spatiotemporal expression patterns regulated by the genetic network T1, and corresponding equations which regulate this pattern formation. (d). When the expression of gene A in the genetic network T1 is described by Eq S2, the relationship between gene A’s stable states and the expression level of gene A in neighboring cells. (e). When the expression of gene B in the genetic network T1 is described by Eq S13, the intracellular gene B’s stable state value corresponds to two genes in neighboring cells with different expressions. (TIF) [file pcbi.1011882.s015.tif]

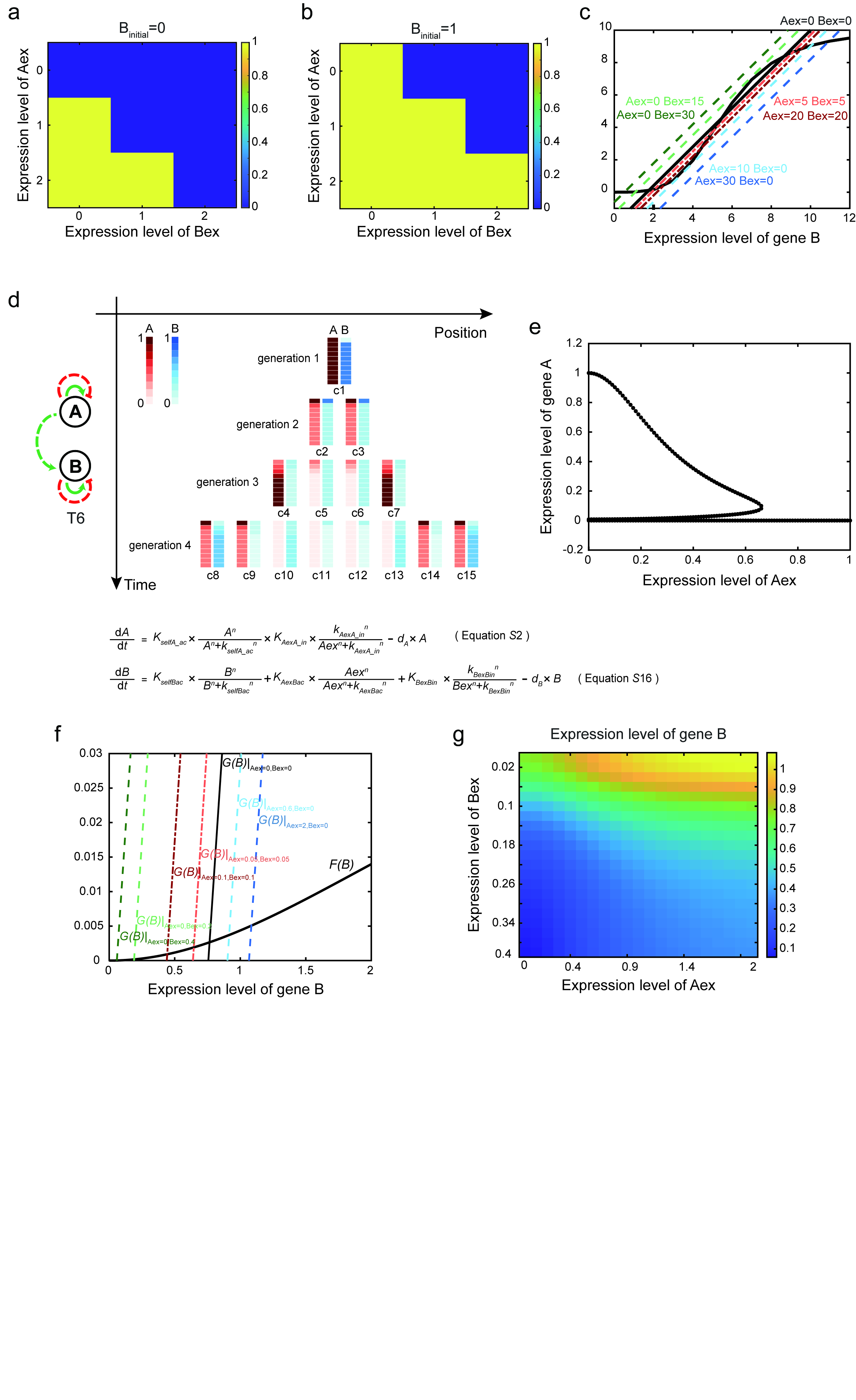

Supplement: S11 Fig — (a). Phase portrait of gene B in the Boolean model corresponding to the genetic network T1. When gene B’s initial expression level is low, the influence of various variables on its stable state. Different colors represent different stable state values of gene B. (b). Phase portrait of gene B in the Boolean model corresponding to the genetic network T1. When gene B’s initial expression level is high, the influence of various variables on its stable state. Different colors represent different stable state values of gene B. (c). Eq S16 may regulate the expression of gene B, resulting in a bistable phase portrait of gene B under a set of particular parameters. Colored dashed lines represent zero-solution lines for various external parameters. (d). The spatiotemporal expression patterns regulated by the genetic network T6, and corresponding equations which regulate this pattern formation. (e). When the expression of gene A in the genetic network T6 is described by Eq S2, the relationship between gene A’s stable states and the expression level of gene A in neighboring cells. (f). The phase portrait of gene B corresponding to Eq S16. Colored dashed lines represent zero-solution lines for various external parameters. (g). When the expression of gene B in the genetic network T6 is described by Eq S16, the intracellular gene B’s stable state value corresponds to two genes in neighboring cells with different expressions (TIF) [file pcbi.1011882.s016.tif]
