# Supplementary material for: A mathematical framework for understanding the spontaneous emergence of complexity applicable to growing multicellular systems
Source: PLoS Comput Biol. 2024 Jun 5;20(6):e1011882. doi: 10.1371/journal.pcbi.1011882 (PMC11182560; doi:10.1371/journal.pcbi.1011882)
Supplement: Attachment — Submitted filename: Response-Letter.docx [file pcbi.1011882.s018.docx]

Paper ID: PCOMPBIOL-D-24-00204

The Author Response for “A mathematical framework for understanding the spontaneous emergence of complexity applicable to growing multicellular systems”

Lu Zhang, Gang Xue, Xiaolin Zhou, Jiandong Huang, Zhiyuan Li

We deeply appreciate the reviewers’ instructive comments and suggestions. We have made every effort to address the raised points comprehensively. Below, we provide detailed responses to each of the reviewers' comments. Our responses are marked in blue, with the text from the manuscript in *italic*.

REVIEWER 1 COMMENTS TO THE AUTHOR WITH RESPONSES

----------

*…Overall, this is an interesting study for understanding mechanisms underlying pattern formation, but I have some confusions on the approach to quantify positional information. I hope that the authors can clarify some of the key concepts in the revision.*

We thank the reviewers for your constructive comment, and agree that a clear comprehension of the key concepts, such as positional information, is crucial for a full understanding of our work. In response to your concerns, we have revised our manuscript to relocate the definitions of critical concepts, including positional information, in order to ensure that the fundamental principles are transparent and accessible to readers.

In our revised manuscript, we articulated that "positional information" is designed to describe both the diversity of cellular states and the reproducibility of the spatial arrangement of different cellular states within a biological system. This concept is bifurcated into two parts. The Shannon entropy of cell states captures the diversity within the system by considering the variety and frequency of different cell states, which are determined by the distinct levels of gene expression. The conditional entropy of cell states quantifies the uncertainty of cell states given their positions, effectively measuring whether cells at a particular position are expressing the correct genes. By subtracting the conditional entropy from the Shannon entropy of cell states, we obtain the positional information, which concurrently measures the diversity and reproducibility of cell states. We hope these revisions will dispel any previous uncertainties and provide a clear, comprehensive understanding of how positional information is quantified within our study.

1. *In Ref 16, quantified positional information refers to how precisely a gene expression pattern can convey information of a cell’s position, given the variability of expression. In this work, it is unclear to me how variability can be generated from deterministic simulations shown in Figs 1 and 2. In the illustration of Fig S1, variability across ‘samples’ would reduce positional information, but it is not clear what ‘samples’ here refer to in the simulations. It seems to me that if the simulations are deterministic, then the ‘positional information’ is simply determined by H(state), which, according to Figs 1 and 2, measures the diversity of the states at a particular time point regardless of cells’ positions. I have a hard time understanding the connection between the ‘positional information’ measured in this study and positional precision that one should be concerned about for development.*

Thank you for your constructive comments and for highlighting the issues regarding the generation of variability in our deterministic simulations and the connection between the positional information within our study and positional precision.

Regarding your first concern, you are correct that the deterministic simulations in Figures 1 and 2 do not produce variability in gene expression levels. In these figures, the expression of each gene is fixed by predefined regulatory rules, resulting in a consistent pattern across simulations; therefore, no variability ("different samples") is generated in these deterministic simulations.

In Figure S1, the term "sample" refers to the outcome of a single simulation run. The left panel of Figure S1 demonstrates the deterministic model where gene expression is regulated without noise, leading to identical outcomes across simulations. In contrast, the right panel of Figure S1 depicts a model with added stochastic effects, where gene expression is influenced by both regulatory rules and random noise, resulting in variability between simulation runs. This variability is represented by different "samples" showing diverse outcomes at the same position across simulations.

Regarding the concept of positional information (*PI*), we clarify that in our study, it is quantitatively defined as $PI\left( state,position \right)=H\left( state \right)-H\left( state | position \right)$ (Equation 4). This equation is composed of two parts: the first term, $H\left( state \right)$, represents the Shannon entropy and measures the diversity of cell states independent of their positions. The second term, $H\left( state | position \right)$, represents the conditional entropy and captures the extent to which the position can predict the state of the cell. This latter term relates to the "positional precision" that you mentioned, reflecting the accuracy of gene expression relevant to the correct positioning of cells during development. Our definition of positional information is similar to that in Reference 16; however, we have utilized it differently by emphasizing the 'difference between cells' rather than focusing on minimizing transmission errors.

Please refer to Page 9-10 of the revised manuscript, lines 271-283 (“*Figure S1a depicts two scenarios of calculating positional information for a simple one-dimensional system with 24 cells, each having 4 potential states. To measure the uncertainty for the two systems we assess the frequency of each state and calculate the total entropy (H(state)=2.00 bit for the left system and 1.98 bit for the right systems). In the left system, since the gene expression levels within cells are rigidly governed by regulatory rules without any variance from internal or external noise, cell states are entirely determined by their positions, resulting in a conditional entropy of 0 bit. Conversely, the right system incorporates variability through both regulatory rules and stochastic influences, manifesting in reduced consistency of cell states at identical positions and a non-zero conditional entropy of 0.68 bit. The positional information for each system is then determined by subtracting the conditional entropy from the total entropy. Consequently, the first system has 2.0 bit of positional information, while the second system has 1.32 bit.*”).

2. *I feel that the lateral inhibition mechanism proposed by the authors have a strong bias towards a ‘salt and pepper’ pattern, and it may reduce positional information in realistic settings. As previously shown (10.15252/msb.20167347), lateral inhibition tends to create a ‘salt and pepper’ pattern where two types of cells are distributed in an intermingled fashion. While this pattern can be beneficial for some tissues, positional information (precision) is presumably low when one looks across multiple samples (embryos), according to the definition in Fig S1. It seems that the authors observed this tendency at the end of the simulation shown in Fig 2d, but they did not comment on this (they also did not comment on the decrease of ‘positional information’ in generation 5, which is another issue to me). This raises concern about the robustness of the pattern. In Fig 1e, for example, if cell 11’s position is taken by a green cell due to cell movement or sporadic gene expression, lateral inhibition would stabilize it, and this would reduce positional information.*

We thank the reviewers for these comments. We defined 'positional information' in our article as the difference between the Shannon entropy of cell states and the conditional entropy given the position of cells. Lateral inhibition can affect both the Shannon entropy and the conditional entropy. A 'salt and pepper' pattern, typically composed of two alternating cell types, maximizes Shannon entropy when the distribution of cell types is even, achieving a theoretical maximum of 2.0 bit. In our simulations within a one-dimensional model with cell divisions, lateral inhibition promoted a more uniform distribution of different cell states, thereby increasing the Shannon entropy of cell states. On the other hand, when the number of cells is fixed, lateral inhibition can maintain multiple multicellular stable states[1]. This suggests that 'salt and pepper' patterns may arise where two cell types are not solely arrayed in a strict one-to-one alternation but also in non-one-to-one configurations, resulting in diverse outcomes across simulations (analogous to different 'samples' depicted in Figure S1). Such variability elevates the conditional entropy, thereby diminishing the positional information within perturbed systems, as highlighted by the reviewer.

Figure 2d results from a deterministic model where the conditional entropy is zero; hence, positional information is determined solely by the Shannon entropy of cell states across all positions. The network corresponding to Figure 2d is the only gene regulatory network from our deterministic model simulations that achieved maximum Shannon entropy (2.0 bit) at a multicellular stable state and includes lateral inhibition motif in its structure. Furthermore, we found a high occurrence rate of the lateral inhibition motif in networks that confer high positional information, suggesting a positive correlation between the presence of lateral inhibition motif and positional information when the system is undisturbed and gene expression is solely determined by regulatory rules.

System growth is another crucial factor we have highlighted that allows our system to get reproducible pattern from the lateral inhibition. When the system developed to the 8-cell stage of generation 4 in Figure 2d, the two central cells do not express either gene A or gene B. According to the gene regulatory rules set forth in the article (Equation 5), these two cells will maintain a state of non-expression for both genes. The progeny cells arising from division of these two cells will inherit the non-expression state from their parent cells and continue to exhibit non-expression of both gene A and gene B. Consequently, from the generation 4 onwards, the number of cells that do not express both gene A and gene B will gradually increase, leading to a growing divergence in the distribution of cell states within the system. The Shannon entropy of cell states will begin to decrease, indicating a reduction in positional information within the system.

Regarding Figure 1e, the shift of cell 11 from non-expression to expression of gene A would indeed reduce positional information. This is because lateral inhibition can stabilize multiple multicellular states when cell numbers are fixed[1]. Therefore, if the system is perturbed and some cell states change, the system may stabilize the new multicellular state rather than revert to the original. As discussed earlier, if the system is affected by internal or external noise after reaching a fixed cell number, lateral inhibition may stabilize an incorrect pattern, thus affecting reproducibility, increasing conditional entropy, and decreasing positional information.

3. *The authors considered the variability of cell cycle length a form of noise. ‘Noise’ is a misleading term to use here, especially in the context of C. elegans, in which cell divisions are asynchronous and precisely controlled.*

We appreciate and agree with the reviewers’ comment. We have revised our terminology to use 'asynchronous cell division' instead of 'noise' to more accurately describe the variation in cell cycle lengths. Please refer to Page 16-17 of the revised manuscript, lines 537-568 (“*Noisy cell state decision involves erroneous gene expression updates with a certain probability (Figure 3f), while differences in cell cycle length contribute to a divergence in the timing of cell divisions, leading to an asynchronous division pattern among cells within the same generation (Figure 3g). We simulate the division-decision system 10,000 times under these two distinct conditions for various genetic networks. Identifying the standard multicellular stable state corresponding to the maximum positional information (2.0 bit) in a noise-free and synchronous environment, we assess how likely systems can reach this state under conditions characterized by stochastic perturbations or asynchrony in cell division. The "accuracy" of the system, determined by the fraction of simulations reaching the standard state given gene expression variability and cell cycle length diversity, decreases for both forms. However, the impact differs slightly: in the case of the first condition, accuracy decreases as the cell cycle length increases, suggesting more mistakes with longer cell cycles. Conversely, for the second condition, where cell division is asynchronous, longer cell cycles enhance accuracy as they provide cells more time to rectify aberrant states. This pattern holds for other screened genetic networks using Equation S1, and T1-regulated systems exhibit better resistance to both conditions than other genetic networks (Figure S8c and S8d).*”). We believe this change enhances the clarity of our findings and thank you for helping us improve our manuscript.

4. *C. elegans embryos undergo asymmetric cell divisions in which cell polarity is determined before division occurs. Even though this pattern formation mechanism may dependent on cell contact. This is conceptually different from what the authors proposed, i.e. each cell divides symmetrically and then integrates the contact information from multiple neighbors for fate decisions. This biological discrepancy needs to be reconciled.*

We thank the reviewers for this comment. We realize the importance of accurately representing the biological processes at play and have amended our manuscript to reflect the correct sequence of events in *C. elegans* development. We now address the asymmetric cell divisions and the predetermined cell polarity before division, and further elucidate how this intrinsic asymmetry, in conjunction with cell-cell contact, influences cell fate decisions. We believe these revisions provide a more precise depiction of the developmental mechanisms in *C. elegans* and align our model more closely with the established biological understanding of this system. Please refer to Page 18-19 of the revised manuscript, lines 686-715 (“*During the incipient stages of C. elegans embryo development, evidence suggests that neighboring cells provide cells with potential information (Figure 5b). Also, the significance of asymmetric cell divisions cannot be ignored, particularly in how cell polarity established prior to division. Our model integrates these elements by accounting for the inherent cell polarity and examining the influence of intercellular interactions on fate determination post-division. Specifically, at the 4-cell stage, cells ABa and ABp, progenies of progenitor cell AB, inherit the same "ab" cell state and equally express the Notch receptor. The anteroposterior disposition of ABa and ABp is contingent upon the polarity that progenitor cell AB establishes before division. Concurrently, the caudal cell P2 expresses the Delta ligand. Prior to the signaling event, the four-cell system exhibits three cell states, representing 1.5 bit of positional information. Meanwhile, cell P2 interacts with cell ABp, but not with cell ABa. Therefore, in a concatenated matrix that includes the states of each cell and their neighboring cells, there is 2.0 bit of potential information. During the signaling event, the Notch-Delta signal pathway prevents cell ABp from activating a specific set of genes associated with the "aba" state. As a result of lacking contact with cell P2, cell ABa transitions from the "ab" cell state to the "aba" cell state. Subsequently, all four cells demonstrate different gene expression states, resulting in the embryo possessing 2.0 bit of positional information. Hence, the spatial contact between cells conveys potential information regarding this event. This also underscores the interplay between intrinsic asymmetric divisions and extrinsic contact-mediated signaling in the developmental process.*”).

5*. Contact dependent cell differentiation and the role of noise in embryonic patterning were extensively studied previously. In the work of Holmes et al. (10.1371/journal.pcbi.1005320), for example, noise was shown to be beneficial for correcting cell fates for organizing patterns. I suggest that the authors discuss some of these related works.*

We thank the reviewer for drawing our attention to this and highlighting the above works. Indeed, this is important information to include in the manuscript and we have expanded our discussion to include a more comprehensive analysis of related works. We now acknowledge that while our current findings emphasize the potential negative implications of noise in reducing positional information, there is substantial evidence in literatures, such as the examples reviewers mentioned, supporting the positive effects of noise in biological systems. Specifically, we have incorporated a discussion on how noise-induced gene expression fluctuations contribute to cellular plasticity during embryonic development, thereby enabling the correction of organizational errors [2]. We also address how noise can facilitate the sharpening of boundaries between adjacent segments through the expression of genes like *hoxb1a*/*krox20* [3]. We believe that these additions provide a balanced view of the dual nature of noise in biological systems, and set the stage for future research directions where we aim to explore the potentially beneficial effects of noise on positional information. Please refer to Page 22 of the revised manuscript, lines 825-838 (“*On the other hand, our current findings indicate that noise-induced fluctuations in gene expression levels can trigger state transitions in cells, reducing the positional information in multicellular stable states within the one-dimensional systems. From this perspective, since multicellular stable states with high positional information have smaller basin sizes, noise can cause the system to escape these states and develop towards others with lower positional information, which is disadvantageous for the system. However, previous studies have confirmed that noise can have not only negative effects on biological systems but also positive ones. For instance, during embryonic development, gene expression noise can enhance the robustness of organization by promoting cellular plasticity, allowing the embryo to correct organizational errors induced by dynamic cellular movements [73]. Moreover, noise in hoxb1a/krox20 expression has been shown to promote the sharpening of boundaries between adjacent segments [74]. Therefore, in future research, we are also interested in exploring the potential beneficial effects of noise on positional information.*”).

6*. Typo in Line 320: a colon is repeated.*

Thanks for spotting this typo. We have amended it in the revised manuscript. Please refer to Page 16 of the revised manuscript, lines 528-530 (“*Therefore, if the number of cells in the system remains constant, randomized initial states tend to evolve into a state with a large basin size, keeping positional information low*.”).

REVIEWER 2 COMMENTS TO THE AUTHOR WITH RESPONSES

-­--­­­­--­-----

*The authors should make clear the parameter values used in simulations of the continuous model, especially those in figure 4. Also, since results are likely to vary with parameter values, the authors should give an indication of how robust the results are, by performing some kind of parameter sensitivity analysis.*

We thank the reviewers for their valuable suggestion. We have included a new section (‘Parameter values in the continuous model’) in the supplementary materials of our manuscript to address your concerns. In this section, we present detailed tables listing the specific parameter values used in the simulations of Figure 4, Figure S10c, and Figure S11d. Furthermore, we have performed a parameter sensitivity analysis in the continuous model. By adjusting the values of each parameter, our analysis identified the upper and lower limits necessary to maintain the development of the system towards a multicellular stable state with maximum positional information. The results of the parameter sensitivity analysis are presented in Table S1 (single-gene systems) and Table S2 (two-gene systems), providing comprehensive information on the range of variation for each parameter.

Table S1. Values of parameters in the continuous model of the single-gene systems and the upper/lower limits of parameter variability.

| Equation S2 (AND-gate) | | | | | |
| --- | --- | --- | --- | --- | --- |
| Parameter | K_selfA_ac_ | k_selfA_ac_ | K_AexA_in_ | k_AexA_in_ | d_A_ |
| Value | 0.14981 | 0.2106 | 1.1816 | 0.752 | 0.1695 |
| Upper Limit of Parameter Increase | 30% | 20% | 30% | 20% | 120% |
| Lower Limit of Parameter Decrease | 50% | 20% | 50% | 10% | 30% |
| Equation S3 (OR-gate) | | | | | |
| Parameter | K_selfA_ac_ | k_selfA_ac_ | K_AexA_in_ | k_AexA_in_ | d_A_ |
| Value | 0.1 | 1.8 | 6.564 | 0.0517 | 6.5867 |
| Upper Limit of Parameter Increase | >200% | >200% | >200% | >200% | >200% |
| Lower Limit of Parameter Decrease | >99% | >99% | 90% | >99% | >99% |
| Equation S4 (constitutive) | | | | | |
| Parameter | K_constitutive_ | | K_AexA_in_ | k_AexA_in_ | d_A_ |
| Value | 0.035 | | 3.406 | 0.1587 | 3.4036 |
| Upper Limit of Parameter Increase | >200% | | >200% | >200% | >200% |
| Lower Limit of Parameter Decrease | >99% | | 70% | 80% | 80% |

Table S2. Values of parameters in the continuous model of the two-gene systems and the upper/lower limits of parameter variability.

| Equation S2 (AND-gate)  Equation S12 (OR -gate) | | | | | | | |
| --- | --- | --- | --- | --- | --- | --- | --- |
| Parameter | K_selfA_ac_ | k_selfA_ac_ | K_AexA_in_ | k_AexA_in_ | d_A_ |  |  |
| Value | 0.0169 | 0.1547 | 4.2664 | 0.3352 | 0.0702 |  |  |
| Upper Limit of Parameter Increase | >200% | 110% | >200% | 110% | 180% |  |  |
| Lower Limit of Parameter Decrease | 96% | 60% | 60% | 80% | 94% |  |  |
| Parameter | K_AexBac_ | k_AexBac_ | K_BexBin_ | k_BexBin_ | d_B_ |  |  |
| Value | 0.021 | 2.663 | 0.3219 | 0.0409 | 0.3218 |  |  |
| Upper Limit of Parameter Increase | >200% | >200% | >200% | >200% | >200% |  |  |
| Lower Limit of Parameter Decrease | >99% | >99% | 94% | >99% | 80% |  |  |
| Equation S2 (AND-gate)  Equation S13 (AND -gate) | | | | | | | |
| Parameter | K_selfA_ac_ | k_selfA_ac_ | K_AexA_in_ | k_AexA_in_ | d_A_ |  |  |
| Value | 0.144 | 0.09 | 0.7717 | 0.3088 | 0.1103 |  |  |
| Upper Limit of Parameter Increase | >200% | >200% | >200% | 50% | >200% |  |  |
| Lower Limit of Parameter Decrease | 70% | 50% | 70% | 70% | 95% |  |  |
| Parameter | K_AexBac_ | k_AexBac_ | K_BexBin_ | k_BexBin_ | d_B_ |  |  |
| Value | 0.1972 | 0.7081 | 2.179 | 0.1156 | 0.2862 |  |  |
| Upper Limit of Parameter Increase | >200% | 140% | >200% | >200% | >200% |  |  |
| Lower Limit of Parameter Decrease | 80% | >99% | 80% | >99% | 80% |  |  |
| Equation S2 (AND-gate)  Equation S16 (OR-gate) | | | | | | | |
| Parameter | K_selfA_ac_ | k_selfA_ac_ | K_AexA_in_ | k_AexA_in_ | d_A_ |  |  |
| Value | 0.144 | 0.09 | 0.7717 | 0.3088 | 0.1103 |  |  |
| Upper Limit of Parameter Increase | 170% | >200% | 170% | 50% | >200% |  |  |
| Lower Limit of Parameter Decrease | 80% | 50% | 80% | 94% | 60% |  |  |
| Parameter | K_selfBac_ | k_selfBac_ | K_AexBac_ | k_AexBac_ | K_BexBin_ | k_BexBin_ | d_B_ |
| Value | 0.0525 | 3.3223 | 0.1 | 0.7081 | 0.2165 | 0.1156 | 0.2862 |
| Upper Limit of Parameter Increase | >200% | >200% | 80% | >200% | >200% | >200% | >200% |
| Lower Limit of Parameter Decrease | >99% | 94% | >99% | 30% | 60% | 60% | 60% |

These findings further confirm our previous results obtained through stability analysis of equations. Specifically, in the single-gene system, only Equation *S*2 can exhibit bi-stability for gene A within a certain range of parameter values, while Equations *S*3 and *S*4 cannot. The presence or absence of bi-stability in gene A leads to the emergence of different multicellular stable states at the 8-cell stage in generation 4. In the two-gene system, to achieve the development of four distinct cell states at the 8-cell stage, gene A must exhibit bi-stability, while there are no restrictions on gene B. When bi-stability of gene A is required, the range of parameter values in the corresponding equations is quite narrow; conversely, when bi-stability is not necessary for gene A, the range of parameter values in the equations is considerably broader.

Please refer to Page 41 of the revised manuscript, lines 1483-1530 (‘Parameter values in the continuous model’ section)

*line 300 strip -> stripe*

*line 310 remove "potentially"*

*line 312 "lead to" -> "represent"*

*line 320 remove 2nd comma at end*

*line 343 a continuous model/ continuous models*

*line 393 bit -> bits*

*line 619 this does not make sense, please re-write*

*line 721 the sum in the brackets is missing g_i^q(t)*

*line 867 continious -> continuous*

Thanks for spotting these typos. We have amended it in the revised manuscript.

*line 300 strip -> stripe*

Please refer to Page 16 of the revised manuscript, lines 505-509 (“*While motif ratios showed no significant correlation with stripe number (Figure S7e), positional information negatively correlated with intercellular direct lateral activation and positively correlated with intercellular direct lateral inhibition motif ratios (Figure S7f).*”).

*line 310 remove "potentially"*

Please refer to Page 16 of the revised manuscript, lines 517-518 (“*The basin size represents the total initial cell states evolving into a stable state under specific genetic network regulation.*”).

*line 312 "lead to" -> "represent"*

Please refer to Page 16 of the revised manuscript, lines 518-520 (“*For instance, in network T1, with two cells, there are 16 initial states, and only 10 represent stable multicellular states, suggesting a mean basin size of 1.6.*”).

*line 320 remove 2nd comma at end*

Please refer to Page 16 of the revised manuscript, lines 528-530 (“*Therefore, if the number of cells in the system remains constant, randomized initial states tend to evolve into a state with a large basin size, keeping positional information low*.”).

*line 343 a continuous model/ continuous models*

Please refer to Page 17 of the revised manuscript, lines 570-571 (“*The principle of increased positional information is applicable to continuous models.*”).

*line 393 bit -> bits*

Please refer to Page 18 of the revised manuscript, lines 678-680 (“*Remarkably, our calculations revealed that the embryo possesses more than 5.0 bits of positional information by the time it reaches the 200-cell stage (See supplement for details).*”).

*line 619 this does not make sense, please re-write*

Thank you for your suggestion. We have revised the figure caption as per your recommendation. Please refer to Page 27 of the revised manuscript, lines 963-970 (“*According to the maximum positional information achievable in the development system directed by genetic networks, networks with the same maximum positional information are grouped together. The first column represents the maximum positional information value for each genetic network group. The second column indicates the number of genetic networks in each group. The third column represents the ratio of direct lateral inhibition motif present in the group. The fourth column represents the ratio of indirect lateral inhibition motif present in the group. The fifth column represents the proportion of networks in the group that do not exhibit lateral inhibition motif.*”).

*line 721 the sum in the brackets is missing g_i^q(t)*

Please refer to Page 7 of the revised manuscript, lines 183-185 (“*The first term in the right side of the bracket (*${\sum_{q=1}^{n} a_{i,i}^{p,q}g}_{i}^{q}\left( t \right)$*) depicts how genes within the i-th cell regulate each other, and* $a_{i,i}^{p,q}$ *represents the genetic network within a cell.*”).

*line 867 continious -> continuous*

Please refer to Page 35 of the revised manuscript, lines 1174-1176 (“*In one-dimensional division-decision systems, the simulation of the continuous model leads to increased positional information (Figure 4a, 4b and 4c).*”).

*The authors should consider if all the supplementary figures are absolutely necessary, as there are very many.*

Thanks for pointing this out. We have carefully reviewed the supplementary figures and have made some adjustments by merging and eliminating redundant figures. As a result, we have reduced the total number of supplementary figures from the original 14 to the current 12.

**We would like to gratefully thank the editor and all reviewers again for their time and effort in reviewing our manuscript, and please feel free to contact us if you have any question.**

REFERENCES

1.	Collier JR, Monk NA, Maini PK, Lewis JH. Pattern formation by lateral inhibition with feedback: a mathematical model of delta-notch intercellular signalling. J Theor Biol. 1996;183(4):429-46. doi: 10.1006/jtbi.1996.0233. PubMed PMID: 9015458.

2.	Holmes WR, Reyes de Mochel NS, Wang Q, Du H, Peng T, Chiang M, et al. Gene Expression Noise Enhances Robust Organization of the Early Mammalian Blastocyst. PLoS Comput Biol. 2017;13(1):e1005320. Epub 20170123. doi: 10.1371/journal.pcbi.1005320. PubMed PMID: 28114387; PubMed Central PMCID: PMCPMC5293272.

3.	Zhang L, Radtke K, Zheng L, Cai AQ, Schilling TF, Nie Q. Noise drives sharpening of gene expression boundaries in the zebrafish hindbrain. Mol Syst Biol. 2012;8:613. doi: 10.1038/msb.2012.45. PubMed PMID: 23010996; PubMed Central PMCID: PMCPMC3472692.
